# Discovery of Novel HDAC8 Inhibitors Through Shape-Based Virtual Screening: Experimental Validation and Characterization of a Promising Quinoline-Based Hit Scaffold

**DOI:** 10.3390/ijms27219557

**Published:** 2026-10-26

**Authors:** Adriana Gargano, Aday González-Bakker, Adrián Puerta, Valentina Maggisano, Stefania Bulotta, Roberta Rocca, José M. Padrón, Stefano Alcaro

**Affiliations:** 1 Dipartimento di Scienze della Salute, Università “Magna Græcia” di Catanzaro, Campus Universitario “S. Venuta”, Viale Europa, 88100 Catanzaro, Italy; a.gargano@unicz.it (A.G.); vmaggisano@unicz.it (V.M.); bulotta@unicz.it (S.B.); alcaro@unicz.it (S.A.); 2 Associazione CRISEA—Centro di Ricerca e Servizi Avanzati per l’Innovazione Rurale, Località Condoleo, 88055 Belcastro, Italy; 3 BioLab, Instituto Universitario de Bio-Orgánica “Antonio González”, Universidad de la Laguna, C/Astrofísico Francisco Sánchez 2, 38206 La Laguna, Spain; agonzaba@ull.es (A.G.-B.); jmpadron@ull.es (J.M.P.); 4 Institute of Biological and Chemical Systems—Functional Molecular Systems, Karlsruhe Institute of Technology (KIT), 76344 Eggenstein-Leopoldshafen, Germany; adrian.puerta@kit.edu

**Keywords:** HDAC8 inhibitors, shape virtual screening, natural compounds

## Abstract

Histone deacetylase 8 (HDAC8) is overexpressed in several solid tumors and, unlike other Class I HDACs, functions independently of large corepressor complexes, making it an attractive target for structure-based drug design. Here, we aimed to identify novel natural compounds capable of targeting the human HDAC8 catalytic site. A library of 307,814 natural products from ZINC15 was screened by shape-based virtual screening using five structurally diverse co-crystallized HDAC8 ligands as templates, followed by molecular docking and Molecular Mechanics-Generalized Born Surface Area (MM-GBSA) binding free energy calculations. Seven commercially available hits were selected for enzymatic validation, leading to the identification of 4-((7-methoxyquinolin-4-yl)amino)benzamide (ZINC000054202375, compound **2**) as a promising HDAC8 inhibitor. In vitro antiproliferative assays across multiple human solid tumor cell lines demonstrated significant growth inhibition by compound **2**, further supported by colony formation assays. Mechanistic studies using label-free live-cell imaging, Annexin V staining, DAPI (4′,6-diamidino-2-phenylindole) nuclear morphology, and DCFDA (2′,7′-dichlorodihydrofluorescein diacetate) assays revealed membrane blebbing, mitotic failure, increased reactive oxygen species, and nuclear fragmentation, consistent with apoptosis induction. Finally, 1 μs molecular dynamics simulations supported the formation of a persistent ligand–receptor interaction network within the HDAC8 catalytic pocket. Overall, compound **2** represents a promising non-hydroxamate hit scaffold with promising HDAC8 inhibitory activity, serving as a starting point for the development of novel HDAC-targeted anticancer agents.

## 1. Introduction

The spatial arrangement of DNA within chromatin plays a crucial role in establishing distinct gene expression patterns. In chromatin, DNA is packaged into nucleosomes, which are composed of DNA strands of around 146 bp wrapped around histone proteins [1,2]. This histone-mediated compaction reduces DNA length to some extent and is critical for the formation of complex chromatin architectures. As the amino-terminal extensions of the four core histones extend from the nucleosome they are subjected to various post-translational modifications, including lysine acetylation, lysine and arginine methylation, serine phosphorylation, and ubiquitination [3,4]. These alterations can function as epigenetic markers, regulating specific gene expression [5].

Histone acetylation, typically associated with transcriptionally active chromatin, enhances the accessibility of DNA to the transcription machinery. Conversely, reduced histone acetylation is associated with transcriptionally inactive, more compacted chromatin regions. Histone modifications are regulated through the coordinated actions of transcriptional activators and repressors, which modulate chromatin structure by recruiting specific modifying enzymes, such as histone acetyltransferases (HATs) and histone deacetylases (HDACs) [5].

HDACs, also known as lysine deacetylases (KDACs), are enzymes that utilize either zinc ions (Zn^2+^) or nicotinamide adenine dinucleotide (NAD^+^) as cofactors [6,7]. By removing acetyl groups from lysine residues in histone tails [8], these enzymes play a pivotal role in modulating chromatin structure and regulating numerous downstream biological signaling pathways. Mechanistically, histone acetylation counteracts the positive charge of lysine residues, neutralizing their interaction with the negatively charged DNA and promoting a relaxed chromatin state. Conversely, HDAC-mediated deacetylation restores these positive charges, leading to a more compacted chromatin structure and reduced gene expression [8]. HDACs and HATs function cooperatively to control acetylation dynamics and preserve epigenetic stability across cellular systems [9]. While HATs introduce acetyl groups onto histone tails, neutralizing their charge and relaxing chromatin structure, HDACs remove these acetyl groups, promoting chromatin compaction and gene repression. Moreover, HDACs regulate gene transcription by deacetylating non-histone proteins [8]. This balance between HAT and HDAC activity is fundamental for the dynamic control of chromatin structure and gene expression. Furthermore, HDACs impact other post-translational modifications (PTMs) such as methylation, ubiquitination, propionylation, butyrylation, formylation, phosphorylation, sumoylation, citrullination, proline isomerization, and ADP ribosylation [10].

The functionality of diverse HDAC isoforms is vital for normal physiological processes; indeed, disruptions in the equilibrium of acetylated status of histones are correlated with numerous pathological conditions, including neurodegenerative and cardiovascular disorders, autoimmune diseases, metabolic disorders, diabetes, and cancer [8].

HDACs are subdivided into 18 isoforms classified into four subfamilies: Class I, Class II, Class III, and Class IV [11,12]. The Class I subfamily, which includes HDAC8, also comprises HDAC1, HDAC2, and HDAC3 [13,14]. These enzymes play critical roles in fundamental cellular processes, including cell proliferation, cell cycle regulation, and apoptosis. HDAC3 is known for its involvement in cell cycle control and the DNA damage response [15,16], while HDAC1 and HDAC2, due to their high homology, share overlapping functions in regulating cell proliferation and apoptosis [17].

Unlike other HDAC isoforms, HDAC8 expression in normal human tissues is primarily confined to cells undergoing smooth muscle differentiation. This includes smooth muscle cells from both visceral and vascular sources, as well as myoepithelial cells and myofibroblasts, with HDAC8 predominantly localized in the cytoplasm [18]. Its enzymatic activity relies on a catalytic pocket containing a critical Zn^2+^ ion coordinated by conserved amino acid residues, which facilitates the nucleophilic attack on the acetylated substrate. This structural arrangement is essential for preserving the differentiation status and proper function of smooth muscle cells, key players in numerous physiological processes [8]. Dysregulation of HDAC8 has been implicated in different pathological conditions, highlighting its critical role in maintaining cellular homeostasis [19].

Histone deacetylase 8 (HDAC8) stands out among Class I HDAC enzymes due to its unique structural and functional features. Unlike its counterparts, it lacks a C-terminal extension and possesses critical catalytic sites, which include zinc ions and arginine residues essential for its enzymatic activity. The catalytic domain, composed of 377 amino acid residues, forms a structure with eight parallel β-sheet strands surrounded by either eleven or thirteen α-helices. This configuration forms a narrow hydrophobic channel shaped by specific amino acid residues, including Phe152, Phe208, His180, Gly151, Met274, and Tyr306. The Zn^2+^ ion at the base of this channel coordinates with His180, Asp178, Asp267, the acetyl group oxygen of the substrate, and a water molecule involved in hydrolysis [20]. This channel accommodates the acetylated lysine side chain, facilitating its deacetylase function. Additionally, two metal ion binding sites within the catalytic domain, designated Site 1 and Site 2, play key roles in enhancing the enzyme’s stability and catalytic efficiency. Site 1 is proximal to the zinc ion binding site, while Site 2 is situated on the periphery of the catalytic domain [8,20].

Histone deacetylase inhibitors (HDACis) exert their effects by modifying histone acetylation levels, resulting in changes to chromatin structure that can reactivate silenced genes and suppress the expression of genes involved in cell proliferation. These agents have shown significant anticancer potential through multiple mechanisms, including the induction of apoptosis, cell cycle arrest, and the promotion of autophagy [21]. Although HDACis encompass structurally diverse classes such as hydroxamic acids, cyclic peptides, and benzamides, they all retain a conserved pharmacophore architecture critical for zinc-dependent catalytic inhibition. This model typically comprises three structural elements: a zinc-binding group (ZBG) that chelates the catalytic Zn^2+^ ion at the active site; a linker region that mimics the natural substrate and occupies the enzyme’s active site tunnel; and a hydrophobic cap group (CAP) that interacts with amino acid residues at the surface of the active site pocket.

Despite promising preclinical and clinical data, HDACis still face several limitations, including poor bioavailability, rapid metabolic degradation, irreversible induction of cell differentiation, and limited selectivity for malignant versus normal cells [8], especially in solid tumors.

Trichostatin A (TSA) was the first naturally occurring hydroxamic acid identified as a potent HDACi, and it served as a structural template for the design of synthetic analogues [22,23,24]. Among these, Vorinostat (suberoylanilide hydroxamic acid, SAHA) emerged as the first HDACi approved by the FDA for the treatment of cutaneous T-cell lymphoma, due to its favourable pharmacological profile and structural resemblance to TSA [25,26,27]. Following SAHA success, several other hydroxamate-based inhibitors have been developed and clinically evaluated [26,28].

Belinostat [28,29] and Panobinostat [30,31], both approved for hematologic malignancies, exhibit broad-spectrum HDAC inhibition with enhanced potency and tolerability [32]. More recently, compounds such as Abexinostat [33] and Quisinostat [34] have been developed to enhance isoform selectivity, minimize off-target toxicity, and improve therapeutic efficacy across both hematologic malignancies and solid tumors [33,35,36]. Furthermore, non-hydroxamate inhibitors, such as Entinostat (a benzamide derivative) [37], are being investigated for their selective inhibition of class I HDACs, potentially offering better safety profiles [38]. Collectively, these developments highlight the ongoing evolution of HDACis from pan-inhibitors to more targeted agents with optimized pharmacodynamic and pharmacokinetic properties.

Despite these advancements, the clinical application of FDA-approved HDACis remains largely restricted to hematological malignancies, while their efficacy as monotherapy in solid tumors has been underwhelming. Nevertheless, the use of HDACis as coadjuvant therapy is emerging as a promising strategy to sensitize solid tumors to conventional treatments, showing significant potential in overcoming resistance to agents such as cisplatin [39].

Given the reported overexpression of class I HDACs in several cancer types, including those of the stomach, esophagus, colon, prostate, breast, and lung [40], and the urgent need for agents capable of exerting robust activity against solid tumors, we carried out a virtual shape-based screening aimed at identifying potential HDAC8 inhibitors. This isoform was specifically selected because, unlike other Class I HDACs that require assembly into larger complexes, HDAC8 is uniquely functional in isolation, making it an ideal target for structure-based design, while its specific oncogenic role in solid tumors offers an avenue to avoid the toxicity associated with pan-HDAC inhibition [8]. Conceived as an early-stage hit-identification study, the primary objective of this work was strictly focused on establishing a novel, non-hydroxamate chemical scaffold and validating its specific ligand–receptor interactions and mechanistic cellular consequences. Consequently, rather than pursuing generic toxicity profiles in standard two-dimensional healthy cell models, which frequently fail to replicate complex in vivo safety windows, this research was designed to systematically map target-driven intracellular pathways. This integrative approach led to the selection of a small set of candidate compounds, which were subsequently validated through enzymatic, molecular dynamics, and cell-based phenotypic evaluations to characterize their inhibitory potential and unique mode of action.

## 2. Results

### 2.1. Molecular Modelling

**Figure 1:**
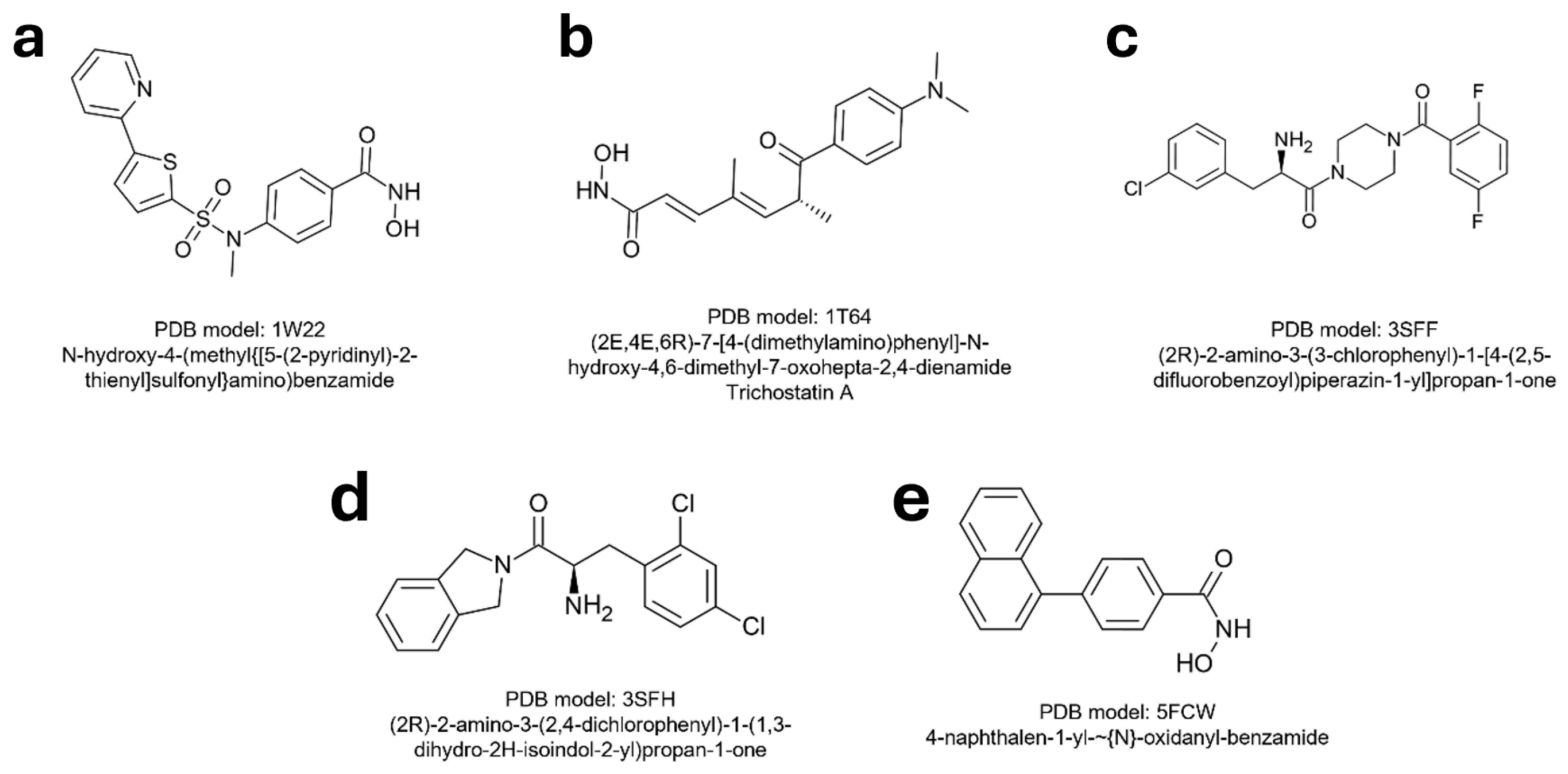
2D chemical structures of ligands co-crystallized with HDAC8 in PDB models: (**a**) 1W22, (**b**) 1T64, (**c**) 3SFF, (**d**) 3SFH, and (**e**) 5FCW. These ligands were selected as query molecules for shape-based virtual screening to identify potential HDAC8 inhibitors.

For our virtual screening campaign, a total of 307,814 natural product-based compounds were retrieved from the ZINC database [41,42]. Through a ligand-based shape analysis, the dataset was refined by retaining only those molecules with a shape similarity score greater than 0.7 when compared to the bioactive conformations of the five selected co-crystallized ligands (Figure 1), ultimately yielding a subset of 9737 candidates. The compounds were docked into the active site of HDAC8 using the crystal structure complexed with the hydroxamic acid ligand, 4-naphthalen-1-yl-(N)-oxidanyl-benzamide (PDB ID: 5FCW) [43]. The docking score of this co-crystallized ligand (−7.18 kcal/mol) was used as a threshold to filter the compounds. Only those with docking scores better than this cut-off proceeded to MM-GBSA binding free energy calculations. Through this virtual screening workflow, 40 candidate compounds were identified, each exhibiting predicted binding energies surpassing that of the co-crystallized reference ligand (−69.56 kcal/mol). Here, the co-crystallized reference ligand was used as a pragmatic energetic benchmark to prioritize compounds from the initial library. This reference-based threshold enabled the selection of candidates with predicted binding energetics comparable to or more favorable than those of the crystallographic ligand for subsequent clustering and selection, while serving as a preliminary filtering criterion rather than a predictor of experimental inhibitory activity [44,45]. These energy values were utilized as an end-state scoring metric to prioritize candidates with favorable predicted steric and electrostatic complementarity for further structural analysis and clustering thereby suggesting a potentially higher binding affinity toward the HDAC8.

To increase the structural diversity among the selected candidates, we computed extended molecular fingerprints and applied clustering analysis based on the Tanimoto similarity coefficient, addressing the high structural similarity observed among several of the identified compounds. One representative ligand from each of the 13 clusters was selected for further evaluation (Figures S1 and S2). From the initial screening set, seven compounds were selected based on favourable docking scores, visual inspection of binding poses, and commercial availability. Their lowest-energy binding conformations were analyzed in detail, and the corresponding docking scores and MM-GBSA binding free energies are summarized in Table 1.

All seven selected hits share a common amide functional group. In most cases, the amide is directly attached to an aromatic ring; however, in compounds **5**, **6** and **7**, it is linked to the aromatic ring via a methoxy spacer. The selected compounds exhibited docking scores (D-Score) ranging from −9.37 to −8.21 kcal/mol, indicating a generally favorable predicted affinity for the HDAC8 binding site. Correspondingly, MM-GBSA binding free energy (ΔG_bind_) values ranged from −82.34 to −70.07 kcal/mol, indicating a relatively consistent and favorable estimated binding energetics across the selected compounds. The differences in ΔG_bind_ may reflect variations in ligand–receptor interaction patterns, binding-site complementarity, and solvation contributions, and were therefore considered primarily for the relative ranking of the compounds rather than as direct measures of complex stability.

**Figure 2:**
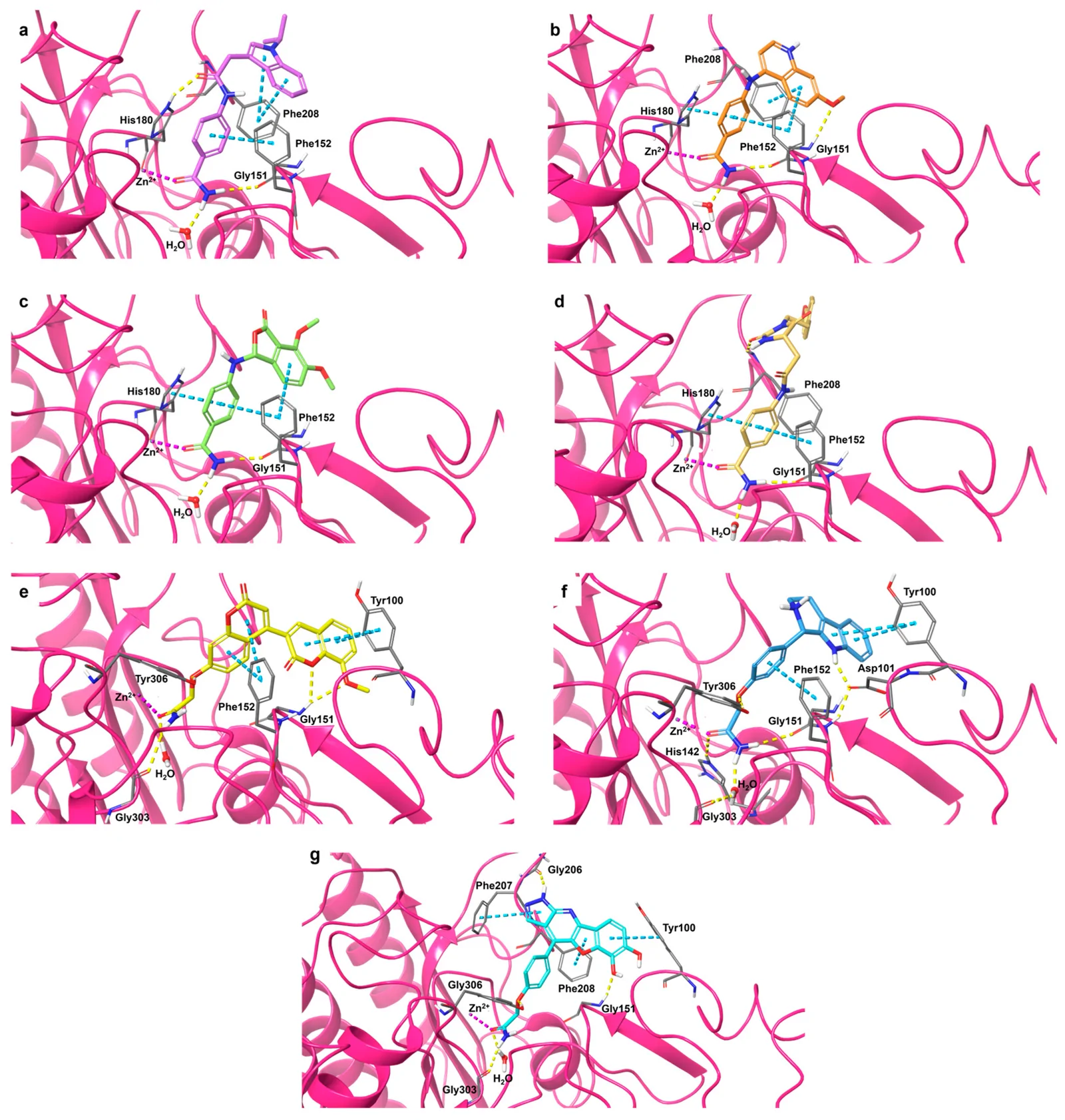
3D representations of the best docking poses of the selected compounds within the HDAC8 binding pocket: (**a**) Compound **1** (faded violet sticks), (**b**) Compound **2** (orange sticks), (**c**) Compound **3** (faded yellow-green sticks), (**d**) Compound **4** (faded orange sticks), (**e**) Compound **5** (yellow sticks), (**f**) Compound **6** (faded azure sticks) and (**g**) Compound **7** (cyan sticks). HDAC8 is shown as a magenta ribbon and key interacting residues are depicted as grey carbon sticks. π–π stacking, electrostatic, and hydrogen bonding interactions are indicated by dashed lines (cyan, pink, and yellow, respectively).

Binding mode analysis of the selected hits indicates that all identified compounds are well accommodated within the enzyme’s active site, establishing interactions with key residues critical for inhibiting its catalytic function. Notably, each compound forms a hydrogen bond (H-bond) with the water molecule involved in hydrolysis, as well as an electrostatic interaction with the Zn^2+^ ion located at the base of the hydrophobic channel (Figure 2).

In particular, compound **1** (ZINC000072325860), which retains the benzamide portion found in previous compounds and forms two π–π interactions with Phe208, one with Phe152, and two H-bonds with Gly151 and His180 (Figure 2a). Compound **2** (ZINC000054202375), which features a methoxyquinoline and a benzamide moiety, is stabilized within the enzyme pocket by multiple π–π interactions with Phe208, His180, and Phe152, along with a H-bond with Gly151 (Figure 2b). A similar interaction pattern is observed in compound **3** (ZINC000032124443), the methoxyquinoline group is replaced by a bulkier, functionalized benzofuranone moiety. This modification results in the loss of the π–π interaction with Phe208 but preserves all other interactions on the opposite side of the molecule (Figure 2c). Compound **4** (ZINC000096114158) shows stabilization of its dioxyindole portion via a H-bond with Phe208, while its benzamide moiety forms two π–π interactions with Phe152 and His180, as well as an additional H-bond with Gly151 (Figure 2d). Compound **5** (ZINC000004340017) interacts with the binding site residues through two π–π stacking interactions with Tyr100, two with Phe152, a H-bond with Tyr306, and additional H-bonds with Gly151 and Gly303 (Figure 2e). Compound **6** (ZINC000020112605) contains a tetrahydropyridinindole moiety stabilized by two π–π stacking interactions with Tyr100 and a H-bond with Asp101. Its phenoxyacetamide moiety additionally forms a π–π interaction with Phe152, several H-bonds with Tyr306, His142, and Gly151, and a water-mediated bridge with Gly303 (Figure 2f). Although structurally distinct from the other products, compound **7** (ZINC000217671196) establishes several similar interactions, including a π–π interaction with Tyr100, H-bonds with Tyr306, Gly303, and Gly151, as well as additional π–π interactions with Phe207, Phe208, and Phe152, and an H-bond with Gly206 (Figure 2g).

### 2.2. HDAC8 Inhibition

**Figure 3:**
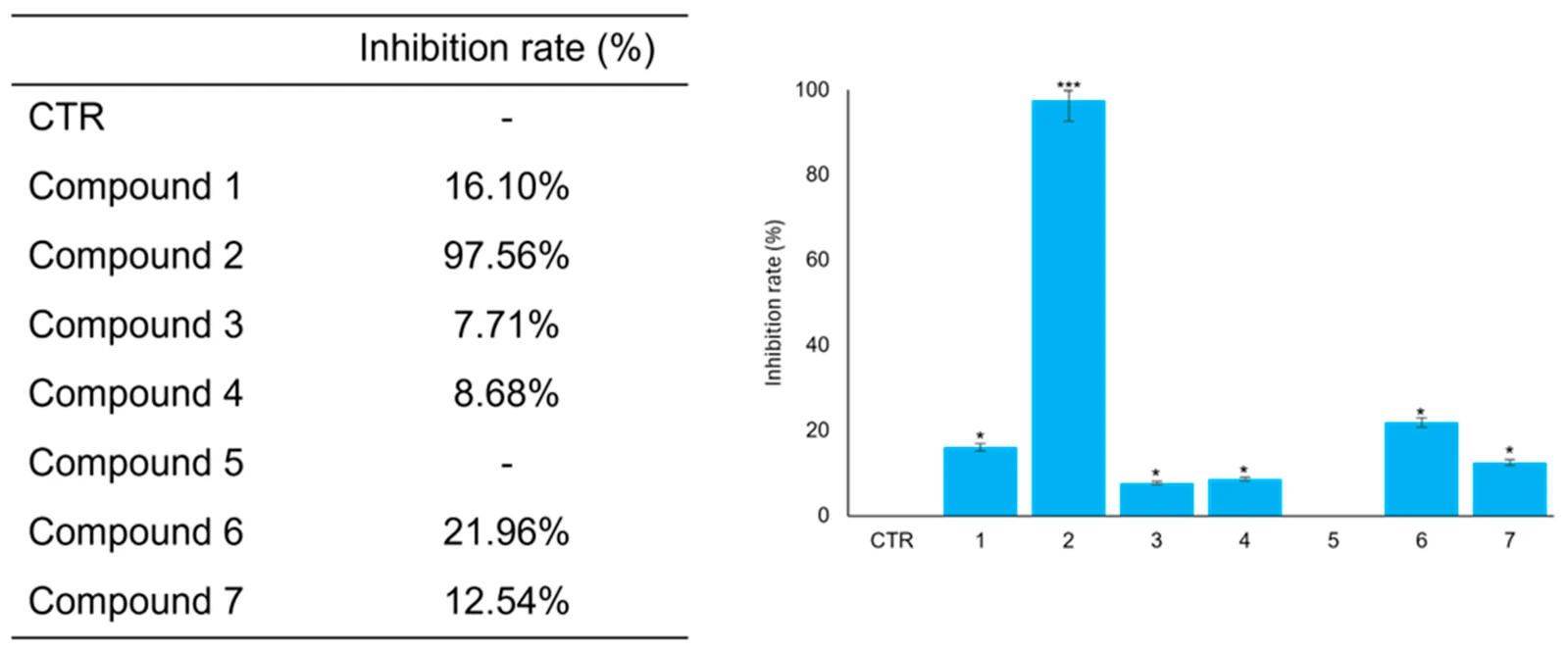
Fluorometric inhibition assay of the selected compounds against HDAC8. Inhibition rate (expressed as %) of the selected compounds against HDC8 enzyme. Data are presented as mean ± standard deviation (*n* = 3). * *p* < 0.05; 0.01, *** *p* < 0.001 vs. CTR.

To validate the in-silico predictions, a fluorometric inhibition assay was conducted. Among the seven compounds selected through virtual screening, five showed limited inhibitory activity, with inhibition values not exceeding 21% at the tested concentrations, whereas one compound showed no appreciable activity. Notably, compound **2** exhibited the highest inhibitory activity, achieving 97.56% inhibition at a single-dose concentration of 1 μM (Figure 3). While a full concentration-response curve to establish an experimental IC_50_ value was not determined in this initial single-dose screening stage, this robust primary response highlights compound **2** as a highly promising hit scaffold for further investigation. This result is consistent with the predicted binding mode and supports the potential of compound **2** as an HDAC8 inhibitor.

### 2.3. Antiproliferative Tests

Following the enzymatic assay results, compound **2** was evaluated for cytotoxic activity by using an MTT assay. Cell viability was assessed based on the reduction of MTT to formazan by metabolically active cells, providing a quantitative measure of the compound’s effect on cell proliferation and survival.

**Figure 4:**
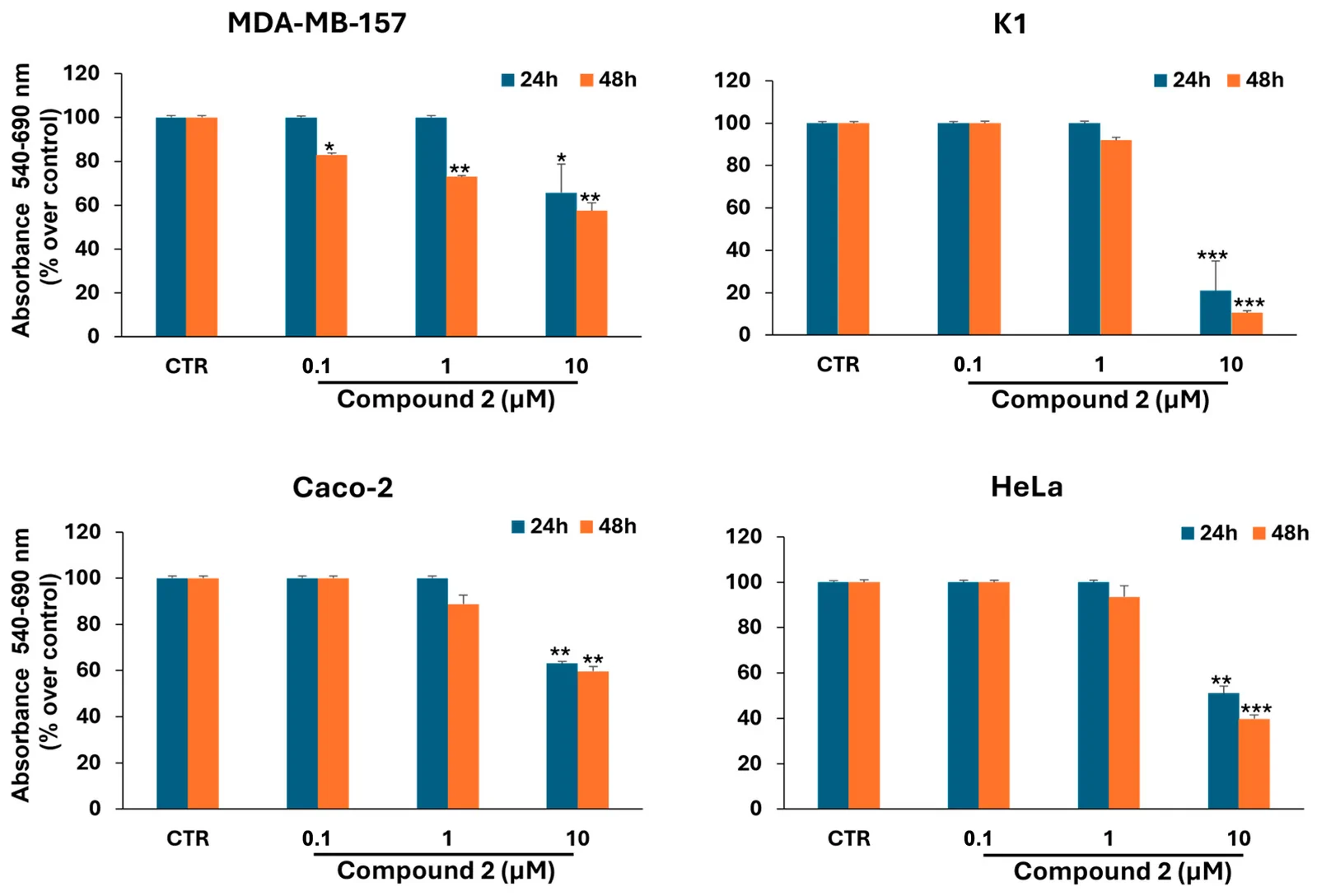
Effect of compound **2** on cell viability. Cell viability was analysed by MTT assay after 24 and 48 h of treatment with various concentrations of compound **2**. Results are expressed as percentage of growth reduction vs. untreated cells (CTR), representing the mean ± SD of three independent experiments. Statistical analysis was performed using the Tukey–Kramer multiple comparisons test. * *p* < 0.05 ** *p* < 0.01, *** *p* < 0.001 vs. CTR.

The graphs in Figure 4 illustrate the percentage of cell growth for four distinct human cancer cell lines, MDA-MB157, K1, Caco-2, and HeLa, at 24 and 48 h post-treatment with a compound. The *X*-axis represents the compound concentration, while the *Y*-axis indicates the percentage of cell growth. The compound demonstrates an inhibitory effect on cell growth across all four cell lines, albeit with varying degrees of sensitivity. Notably, MDA-MB-157 cells showed a dose- and time-dependent response to compound **2**, with a significant reduction in viability at 10 µM after 48 h (~50% of growth inhibition vs. untreated cells, *p* < 0.01). K1, Caco-2, and HeLa cells displayed lower sensitivity to the compound at 0.1 µM and 1 µM, showing almost no impact on viability at 24 and 48 h. However, at 10 µM, a significant reduction in cell growth was observed, with the strongest effect at 48 h leading to 90%, 40%, and 60% growth inhibition, respectively (*p* < 0.001, *p* < 0.01, *p* < 0.001).

Results are expressed as percentage of inhibition vs. untreated cells, representing the mean ± SD of three independent experiments.

To extend the antiproliferative profiling, compound **2** was studied in a representative panel of human solid tumor cell lines. As a model for the antiproliferative activity, the following human solid tumor cell lines were used: A549, HeLa, MIA PaCa-2, SW1573, T-47D, and WiDr. In alignment with early-stage phenotypic drug discovery strategies, our biological evaluation was strictly designed to contextualize the compound’s specific mode of action and intracellular downstream targets (such as target-driven growth inhibition, mitotic failure, and apoptosis) rather than performing broad cytotoxicity screenings. Simple two-dimensional (2D) monolayer cultures of non-tumorigenic cell lines were intentionally excluded from this initial hit-identification phase due to well-documented methodological limitations. Commercially available ‘normal’ cell models require chemical, genetic, or viral immortalization that inherently alters cell cycle regulation and metabolic pathways, often resulting in semi-transformed, tumor-like behaviors. Furthermore, simple 2D monolayers fail to recapitulate crucial in vivo physiological complexities, including three-dimensional tissue architecture, cellular heterogeneity, and microenvironmental interactions, which render early 2D selectivity indexes poorly predictive of actual safety windows. Therefore, the in vitro antiproliferative activity was evaluated after 48 h of drug exposure using the SRB assay. The results expressed as GI_50_ are shown in Table 2.

### 2.4. Clonogenic Assay

Given the time dependency observed of the antiproliferative activity of compound **2**, a clonogenic assay was conducted to evaluate the long-term proliferative potential of HeLa cells after exposure to the hit at varying concentrations. Cells were exposed to either a low dose (LD, 3 μM) or a high dose (HD, 8.8 μM) of the compound for 7 days, and their ability to form colonies was assessed.

**Figure 5:**
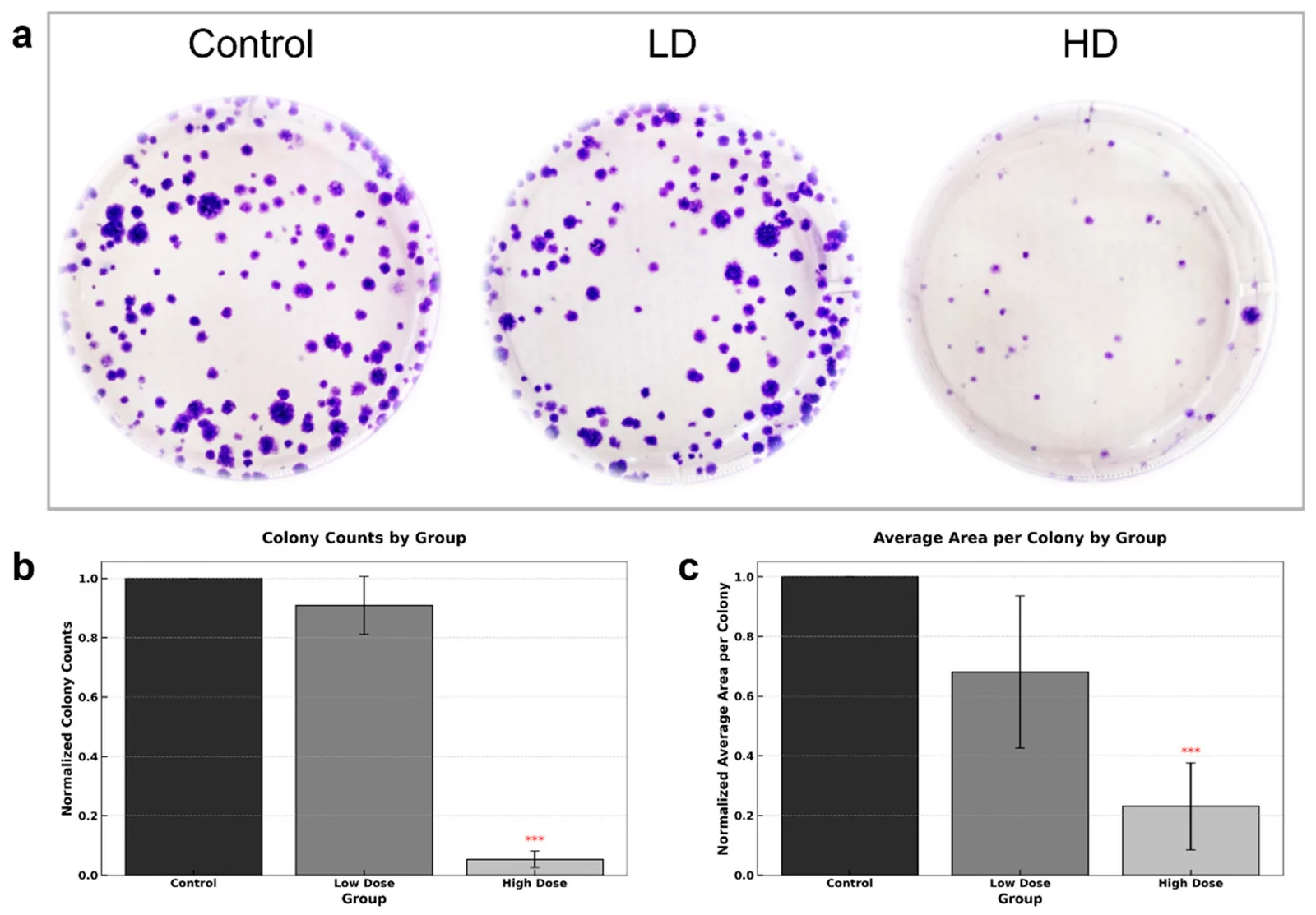
Results of a clonogenic assay on HeLa cells after 7 days of treatment with compound **2**. (**a**) Colony formation under control, low dose (LD, 3 μM), and high dose (HD, 8.8 μM) compound conditions. (**b**) Bar graph quantifying the normalized number of colonies per group. (**c**) Bar graph showing the normalized average area per colony per group. Data represent the mean ± SD of *n* = 3 independent biological experiments, with each experiment carried out in technical triplicate. Statistical analysis was performed using one-way ANOVA followed by Tukey’s multiple comparisons test. *** *p* < 0.01 vs. Control.

The results, as depicted in Figure 5a, clearly demonstrate a dose-dependent reduction in colony formation. The control group exhibited a high density of well-defined colonies, indicating robust cell proliferation. The LD group showed a slight decrease in both the number and size of colonies. In contrast, the HD group demonstrated a significant reduction in colony number and a marked diminishment of colony size was observed.

Quantitative analysis of the colony formation (Figure 5b) confirmed these observations. The normalized colony counts revealed a substantial decrease in the HD group compared to both the control and LD groups. Similarly, Figure 5c illustrates a significant reduction in colony size with increasing compound concentration. Notably, the HD group showed a pronounced decrease in average colony area, supporting a sustained inhibitory effect on cell growth and aggregation.

The finding that the high dose, 8.8 µM, resulted in a substantial reduction in both colony number and size is consistent with the compound’s known growth-inhibitory properties in HeLa cells. The LD, representing a sub-GI_50_ concentration, also demonstrated a reduction in colony formation, although to a lesser extent.

### 2.5. Live Cell Imaging

**Figure 6:**
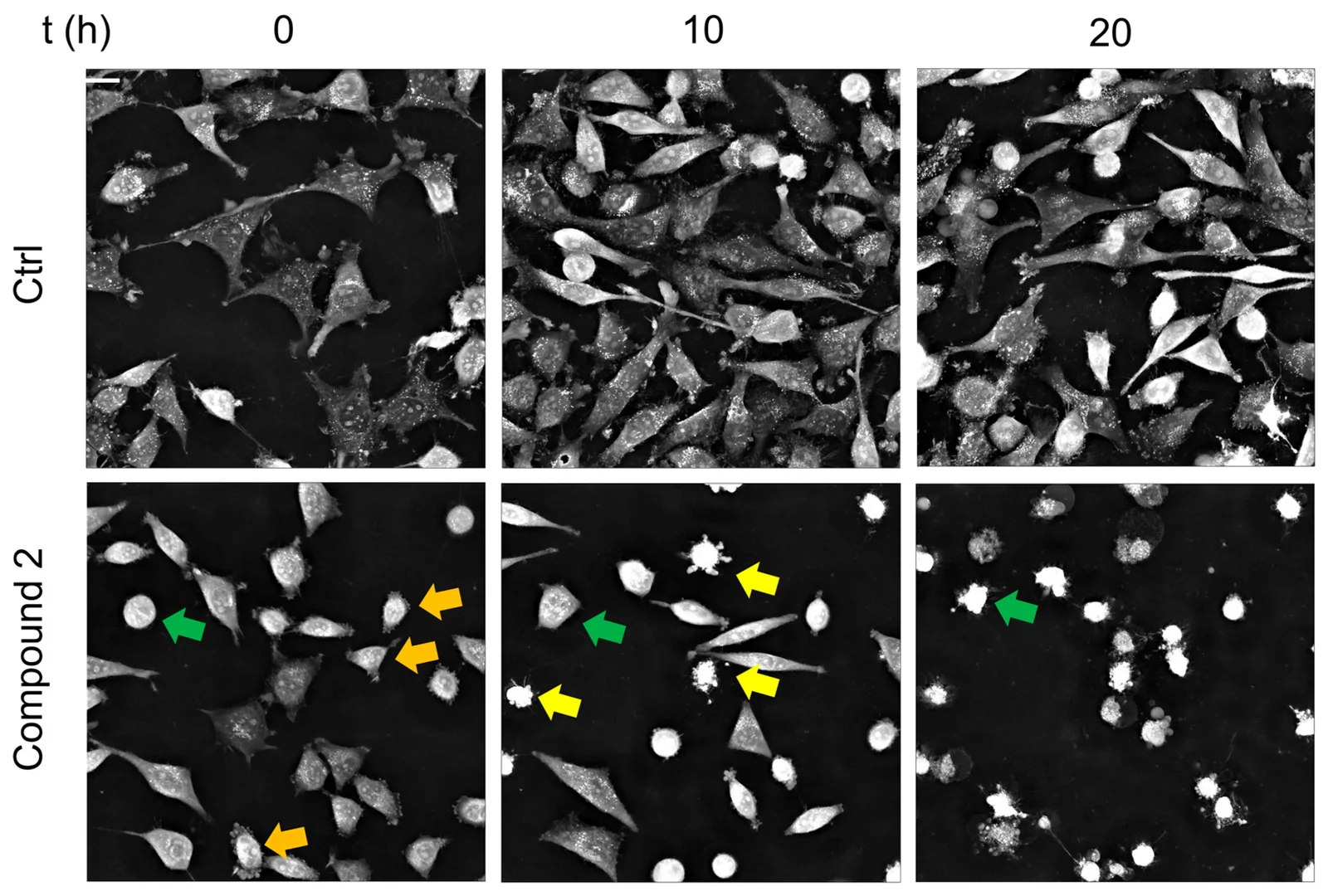
Representative images showing compound **2** dynamic effects on HeLa cells when exposed to 40 µM of the compound. Green arrows mark one cell undergoing aberrant cell division and apoptosis. Orange arrows mark cells presenting reduced area and membrane blebbing. Yellow arrows indicate cells with apoptotic phenotypes. Scale bar 20 µm.

Live-cell imaging provides detailed insights into temporal cellular responses to bioactive compounds through time. Thus, label-free continuous live-cell imaging was performed to evaluate the dynamic behaviour of HeLa cells when treated with the selected candidate (Figure 6 and Supplementary Videos S1 and S2). Cells treated with 40 μM of compound **2** showed a reduction in cell growth compared to untreated samples. It was also observed that early after the addition of the candidate cells appeared shrunk and with observable membrane blebbing (Figure 6, bottom panel), indicating rapid cell death induction upon acute exposure. After 10 h, a significant portion of the imaged cells exhibited features typical of apoptosis, including cellular shrinkage cytoplasmic condensation and formation of apoptotic bodies (Figure 6, bottom panel). By 20 h, widespread cell death was observed across the treated population. Interestingly, dividing cells at early stages of imaging completed unsuccessful mitosis (cytokinesis) where both daughter cells fused together prior to apoptosis induction (an example is marked in Figure 6 with green arrows). This suggests that the candidate affects cells in mitosis as well as interphase cells. This aligns with the described features of HDAC inhibitors that can affect the cell cycle through the downregulation of various key proteins in this process [46].

### 2.6. Apoptotic Induction

**Figure 7:**
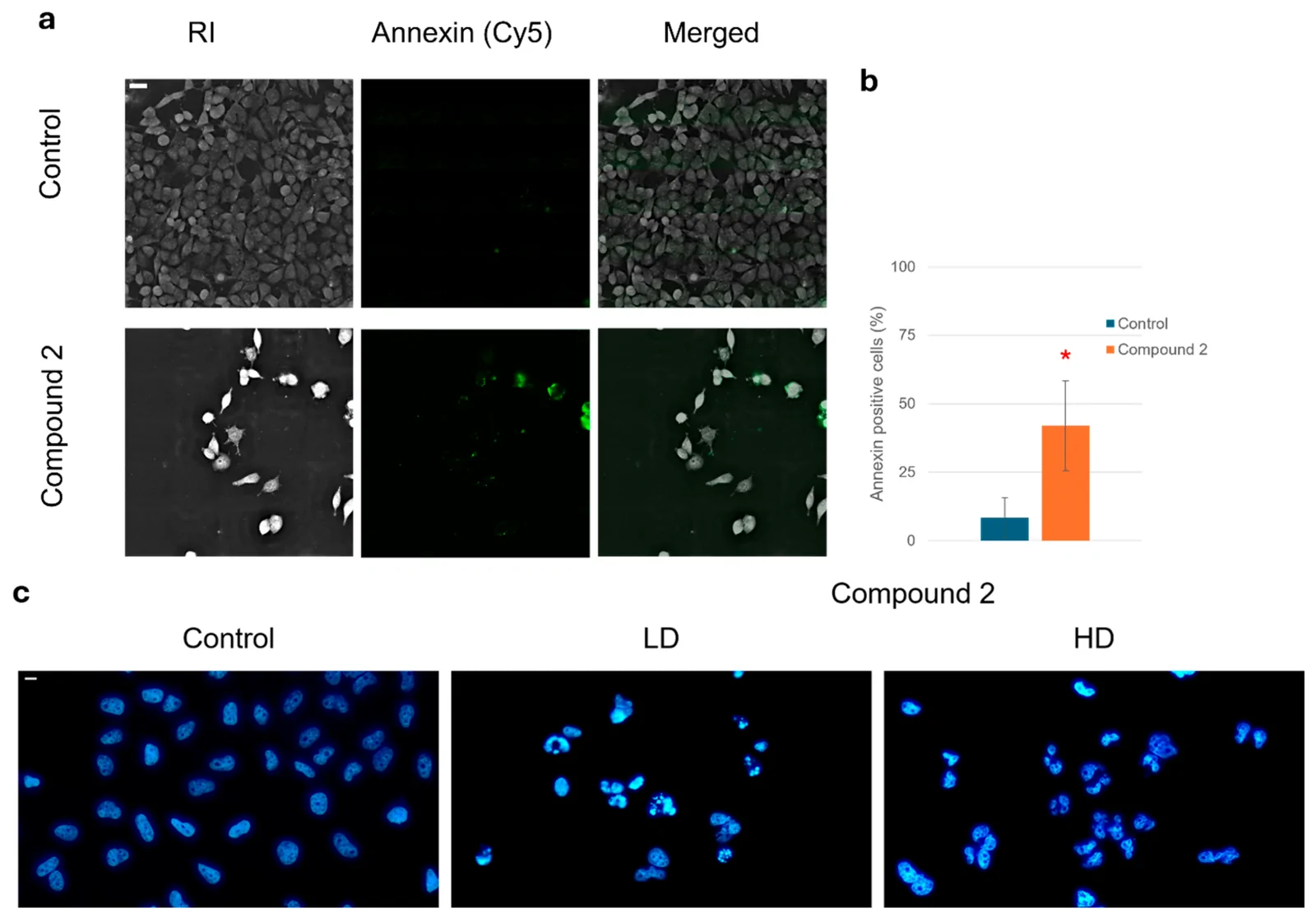
(**a**) Representative refractive index images (RI) and fluorescence (Cy5) images of HeLa cells exposed for 24 h to compound **2** at a concentration of 40 µM and then stained with Annexin V. Scale bar 20 µm. (**b**) Graphical representation of Annexin V-positive cells. Values reflect the mean ± SD of *n* = 3 independent biological experiments. Statistical significance was calculated using a two-tailed Student’s *t*-test. * *p* < 0.05. (**c**) Representative images of DAPI-stained HeLa cells after 24 h of exposure to 20 µM (LD) and 40 µM (HD) of compound **2**. Scale bar 20 µm.

To confirm apoptosis induction, cells were stained with Cy5 conjugated annexin V after 24 h of exposure to compound **2** (Figure 7). Annexin V binds to phosphatidylserine when it is present on the external side of cellular membranes because of apoptosis induction while in healthy cells phosphatidylserine faces the internal side of the cell membrane and Annexin V cannot bind [47]. It was observed that after 24 h of treatment with compound **2** (40 μM), the remaining cells were Annexin V-positive, presenting a fluorescent signal around the cell perimeter. This structural presentation of Annexin V-positivity, combined with the observed dynamic morphological changes, is highly consistent with an apoptotic-like phenotype induced by the candidate compound. In a separate experiment, we sought to examine the compound’s ability to induce nuclear damage and apoptosis hallmarks. To achieve this, HeLa cells were treated with both a low dose (LD) of 20 µM and a high dose (HD) of 40 µM of the compound, and DAPI staining was performed. The rationale for this 24-h time point was to assess longer-term effects on nuclear morphology and to detect potential hallmarks of apoptosis, in addition to the live cell imaging and annexin stain.

Untreated cells presented rounded nuclei with homogeneous staining, whereas multiple treated cells displayed altered nuclear morphology (Figure 7c), including nuclear shrinkage and the formation of apoptotic bodies. These features represent classic morphological hallmarks that are highly consistent with the induction of an apoptotic cell death process. Interestingly apoptotic features were more pronounced at low-dose treatment (LD), showing more condensed nuclei and clear apoptotic bodies. In the high-dose treatment (HD), nuclei appeared with irregular shapes.

### 2.7. DAPI Nuclear Staining Assay

To investigate the potential effects of the compound on nuclear structure and chromatin organization, DAPI staining was conducted at earlier time points. HeLa cells were treated with 40 µM of compound **2**, and DAPI staining was performed at 2 and 4 h post-treatment. This time-course analysis aimed to explore early changes in nuclear morphology prior to the onset of apoptosis.

**Figure 8:**
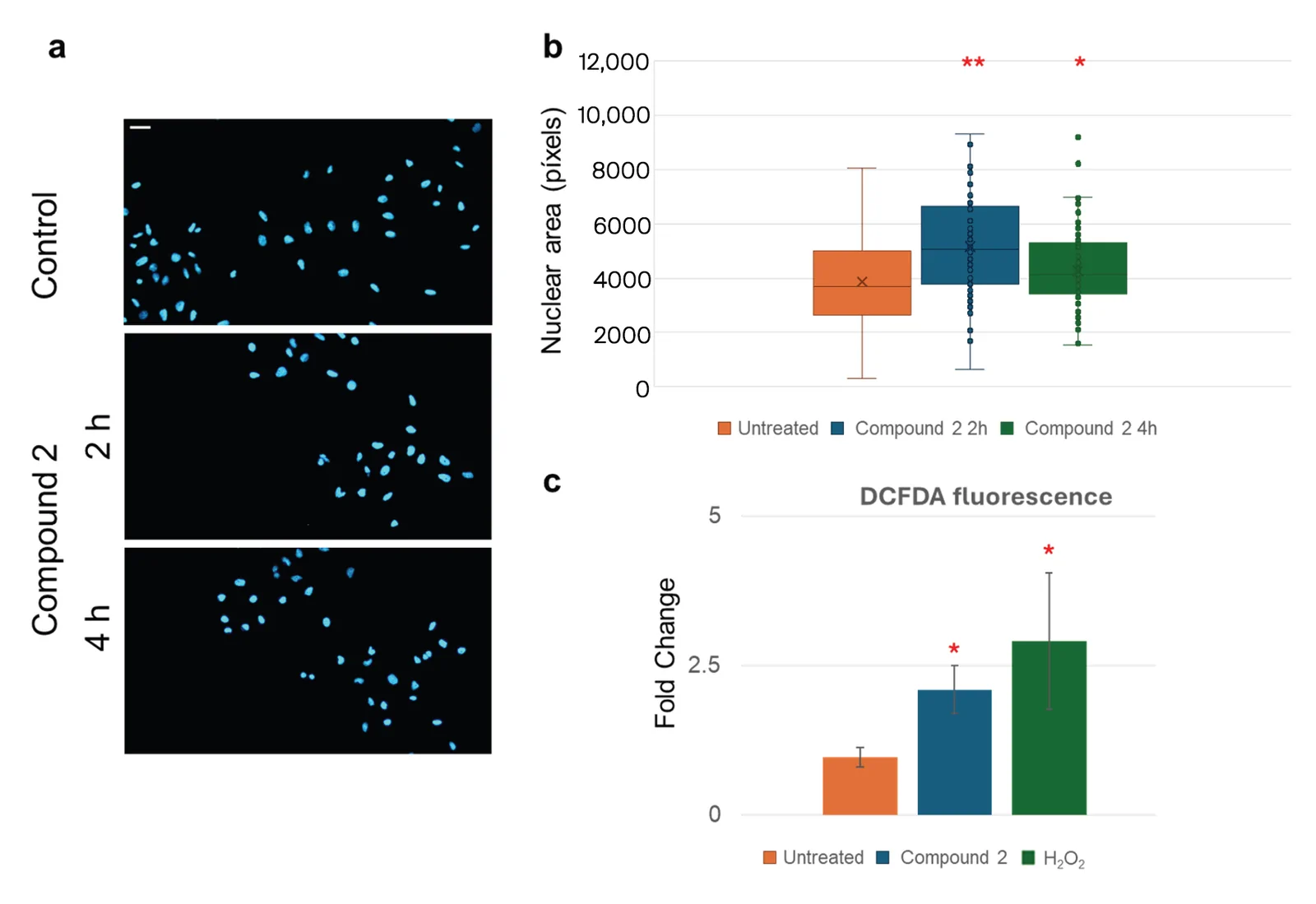
Analysis of nuclear area and ROS formation in HeLa cells treated with compound **2**. (**a**) Representative images (20X) of DAPI-stained cells showing nuclei under control conditions, 2 h post-treatment, and 4 h post-treatment. Scale bar 20 µm (**b**) Quantitative analysis of nuclear area, demonstrating a significant increase in nuclear area at 2 and 4 h compared to the untreated control. (**c**) Graphical representation of DCFDA fluorescent signal of HeLa cells after 4-h exposure to compound **2** (40 µM) or H_2_O_2_ (10%). All data are presented as the mean ± SD of *n* = 3 independent biological experiments, consisting of three technical replicates per run. Statistical analysis was conducted via one-way ANOVA followed by Tukey’s post-hoc multiple comparisons test. * *p* < 0.05, ** *p* < 0.01.

The representative images of DAPI-stained cells (Figure 8a) clearly demonstrate an increase in nuclear size at both 2 and 4 h compared to the untreated control. This observation was further corroborated by quantitative analysis of nuclear area (Figure 8b) revealing a statistically significant increase in nuclear area at both 2 and 4 h, with the 4-h time point exhibiting the most pronounced alteration.

This significant transient expansion of the nuclear architecture represents a phenotypic response that is morphologically comparable to chromatin alterations frequently described upon treatment with established HDAC inhibitors like TSA or Entinostat [48,49]. While DAPI profiling does not directly evaluate histone acetylation levels, this morphological shift occurs prior to the onset of nuclear fragmentation, indicating an early cellular response to compound **2**. Accumulation of reactive oxygen species (ROS) is a well-established downstream mediator of the antitumor activity of HDACis [50]. To study the effect of compound **2** on ROS levels, a DCFDA fluorescent probe was used. Cells were preincubated with DCFDA and exposed for 4 h to 40 µM of compound **2**, H_2_O_2_ (10%) was used as positive control. Measurement of DCFDA fluorescence intensity (Figure 8c) revealed a significant increase in ROS levels in compound **2**-treated cells, which is consistent with HDAC inhibitor-associated apoptotic phenotypes.

Additionally, we studied the effects of compound **2** on cell motility through in vitro wound healing assay in A549 cells; however, no significant reduction in wound closure was observed (Figure S3), suggesting that the primary bioactivity of the compound is driven by growth inhibition and cytotoxicity rather than anti-migratory effects.

### 2.8. Molecular Dynamics

To elucidate the structural basis of the inhibition exhibited by compound **2**, we performed a 1 μs molecular dynamics (MD) simulation of HDAC8 bound to the compound, comparing its dynamics to the co-crystallized ligand in PDB 5FCW. This analysis assessed protein backbone stability, ligand binding behavior, and residue-level flexibility to reveal the molecular determinants of inhibitor interaction.

Root Mean Square Deviation (RMSD) analyses of the protein backbone were performed to assess the conformational stability of HDAC8 (Figure S4). Both HDAC8–compound **2** and HDAC8–co-crystallized ligand complexes reached equilibration within the first 200 ns; however, notable differences emerged thereafter. The HDAC8–compound **2** complex remained highly stable, with an average RMSD below 2.0 Å, indicating that the backbone conformation was well preserved throughout the simulation. In contrast, the HDAC8–co-crystallized ligand complex displayed a higher baseline RMSD and underwent a conformational shift between 500–600 ns, reaching a new, elevated plateau. These findings indicate that the HDAC8 backbone maintains lower average structural fluctuations during the simulation when bound to compound **2** compared to the co-crystallized ligand complex. In parallel, the RMSD of both ligands within the HDAC8 binding site was monitored over the full 1 μs simulation to evaluate their positional stability within the active site.

Ligand RMSD analysis within the HDAC8 binding site revealed marked differences in stability (Figure S5). The co-crystallized ligand (red line) showed high mobility, with a conformational shift around 550 ns leading to increased RMSD and fluctuations, indicative of a flexible binding pose. In contrast, compound **2** (blue line) remained highly stable throughout the 1 μs simulation, reflecting persistent interactions that maintain the ligand within the catalytic site. These results, consistent with the backbone analysis, support a persistent and energetically favorable binding mode for compound **2**.

**Figure 9:**
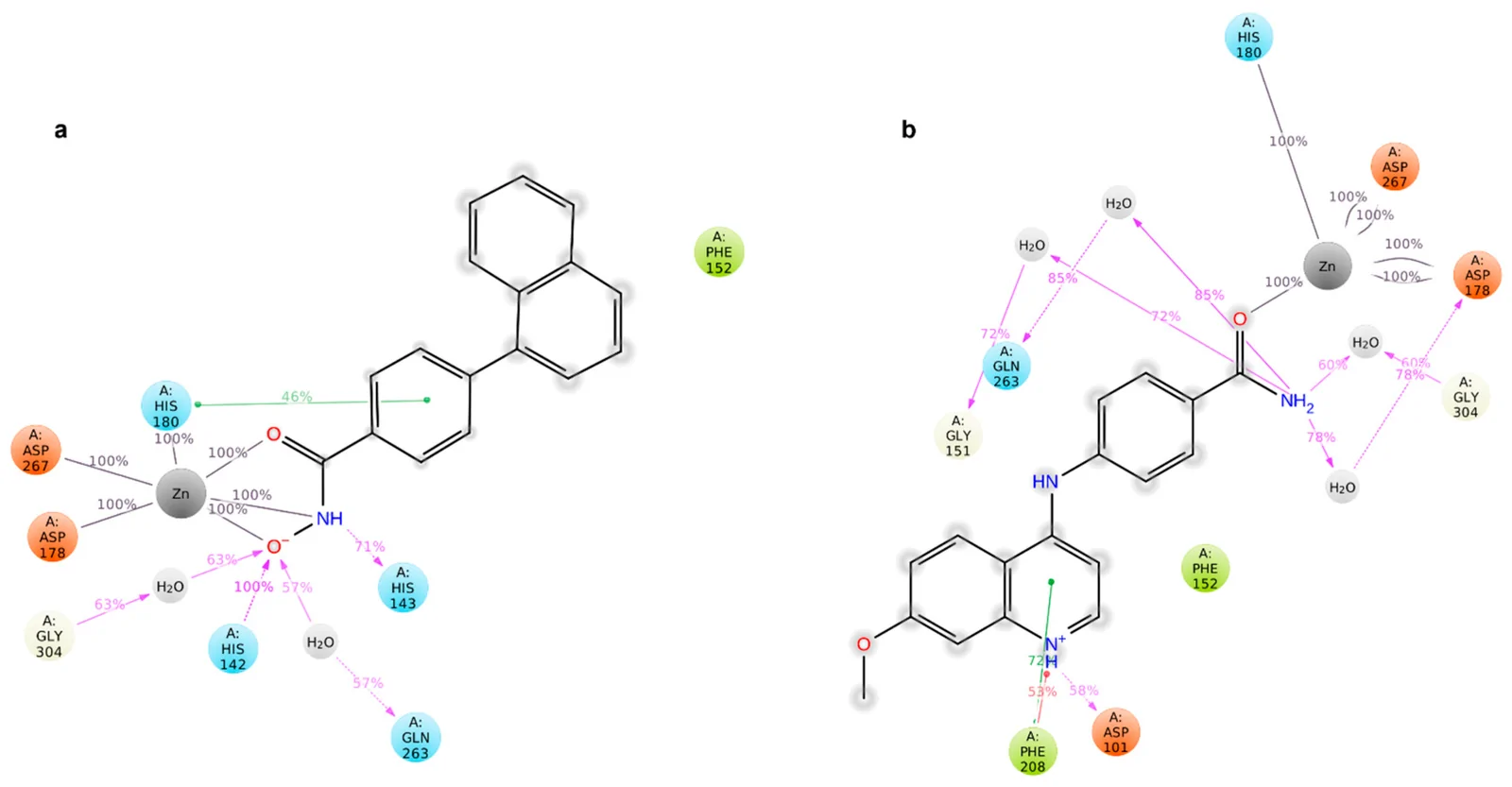
Detailed two-dimensional views of the interactions between the ligands and protein residues during MD simulations. The diagrams show the contact profiles for (**a**) the co-crystallized ligand and (**b**) Compound **2**.

Moreover, Root Mean Square Fluctuation (RMSF) analysis of HDAC8 residues provided insights into local backbone flexibility in the two ligand complexes (Figure S6). The HDAC8–compound **2** complex displayed a consistently lower RMSF profile across several regions, including loops and segments around residue 200 as well as the C-terminal region, indicating reduced local conformational mobility throughout the trajectory compared to the co-crystallized ligand complex. These trajectory-derived metrics indicate that the binding pose of compound **2** remains structurally persistent within the active site, rationalising its experimentally observed in vitro inhibitory activity. Analysis of key interactions during the trajectory further highlighted the structural features of the binding mode (Figure 9). Notably, both the co-crystallized ligand and compound **2** sustained a persistent (100% occupancy) coordination bond with the catalytic Zn^2+^ ion over the 1 µs simulation window. However, compound **2** formed a more extensive and stable network of interactions with key residues, HIS 180, GLN 283, GLY 151, ASP 267, and ASP 178, while the co-crystallized ligand exhibited lower-occupancy bonds with these residues. These results indicate that compound **2** maintains a persistent interaction network within the active site throughout the 1μs trajectory, supported by multiple high-occupancy contact points, thus providing a molecular rationale for its observed HDAC8 inhibitory activity.

In this study, we identified natural HDAC8 inhibitor candidates through a Shape Virtual Screening approach, followed by in vitro validation. Among the selected compounds, compound **2** exhibited remarkable inhibitory activity, achieving a 97.56% inhibition rate at 1 µM in enzymatic assays. Molecular docking and MD simulations indicated that compound **2** establishes persistent interactions within the HDAC8 catalytic site, engaging key active site residues through hydrogen bonding, hydrophobic contacts, and Zn^2+^ coordination.

In vitro experiments confirmed its cytotoxic activity, demonstrating a dose-dependent reduction in viability across multiple cancer cell lines. The clonogenic assay provides compelling evidence for the compound’s ability to inhibit HeLa cell proliferation in a dose-dependent manner. The observed reduction in both colony number and size underscores the compound’s efficacy as a potential anti-cancer agent. Live-cell imaging and fluorescence-based apoptosis assays provided further insights into its mechanism of action, highlighting features such as nuclear fragmentation, membrane blebbing, and apoptotic body formation. Additionally, compound **2** appeared to interfere with proliferation mechanisms, as observed in time-lapse imaging of mitotic cells undergoing unsuccessful division prior to apoptosis.

DAPI staining experiments provide valuable insights into the compound’s effects on nuclear morphology and its ability to induce apoptosis. The time-course study demonstrates early, visible changes in nuclear area that are phenotypically consistent with a relaxed chromatin architecture, a morphological feature described during pathway modulation by classic epigenetic agents. The 24-h study confirms the compound’s ability to induce apoptosis, with distinct morphological features observed at different doses.

The MD trajectory analysis indicates that the proposed binding pose of compound **2** remains persistent throughout the 1μs simulation window, providing qualitative structural context for the docking model alongside a reduction in local per-residue fluctuations (RMSF) in key pocket-lining loops. Overall, these findings present compound **2** as a promising small-molecule scaffold for HDAC8 inhibition, demonstrating characteristic HDAC inhibitor-associated cytotoxic and apoptotic responses in solid tumor models.

## 3. Discussion

The identification of effective small molecules capable of effectively targeting HDAC8 remains a major focus in cancer epigenetics. In this study, we employed an integrated computer-aided drug design workflow, combining ligand-based Shape Virtual Screening, molecular docking, and MM-GBSA binding free energy calculations, which led to the identification of compound **2** (4-((7-methoxyquinolin-4-yl)amino)benzamide) as a promising novel scaffold with remarkable HDAC8 inhibitory activity (97.56% at 1 µM). Notably, compound **2** possesses a benzamide-like zinc-binding group (ZBG) instead of the conventional hydroxamate moiety. Given the well-known severe toxicity and poor pharmacokinetics often associated with hydroxamic acid derivatives, the inhibitory activity of this non-hydroxamate hit represents a significant pharmacophore advantage for future drug development [51,52].

The primary enzymatic screening identified compound **2** as a promising novel HDAC8 inhibitor, providing experimental validation of the computationally guided selection strategy. These findings support the proposed binding mode of compound **2**, while acknowledging that computational descriptors should be interpreted as qualitative structural and energetic evidence rather than direct predictors of experimental potency. The experimentally observed activity of compound **2** is further supported by the structural model and the extended 1 μs molecular dynamics (MD) simulations, which provided insights into the interactions potentially contributing to its inhibitory activity. Throughout the simulation, the ligand maintained stable coordination with the catalytic metal ion and established a persistent network of hydrogen-bonding and hydrophobic interactions with key residues lining the binding pocket. Crucially, comparative trajectory analysis showed that compound **2** maintains a significantly lower RMSD profile and reduces per-residue backbone flexibility (RMSF) compared to the original co-crystallized hydroxamate ligand. These trajectory-derived metrics support the structural persistence and contact stability of the predicted binding pose within the active site over time. Biological evaluation across a panel of solid tumor cell lines, including lung, breast, cervical, colon, thyroid, and pancreatic cancers, showed that compound **2** exerts significant antiproliferative effects across diverse tumor models, with GI_50_ values ranging from 8.8 µM to 16 µM. Furthermore, long-term clonogenic assays demonstrated a significant, dose-dependent decrease in both colony number and average colony size, confirming that compound **2** effectively suppresses long-term proliferative potential and survival in HeLa cells. While mechanistic profiling using live-cell imaging, Annexin V staining, DAPI nuclear morphology assays, and DCFDA measurements revealed that compound **2** triggers hallmarks consistent with HDAC inhibitor-associated apoptotic cell death, a direct causal link between intracellular HDAC8-exclusive engagement and the observed cell death endpoints remains to be formally established. Continuous label-free live-cell imaging provided direct visual evidence of acute cellular responses, including rapid cell shrinkage, membrane blebbing, and the formation of apoptotic bodies. Interestingly, time-lapse videos captured dividing cells attempting cytokinesis but failing, leading to daughter cell fusion prior to apoptosis. This observation is consistent with the known role of HDACis in perturbing cell cycle progression and mitotic spindle assembly through the altered acetylation of non-histone substrates and key cell cycle regulators [46]. Furthermore, early-time-point DAPI assays revealed a statistically significant expansion of the nuclear area at 2 and 4 h post-treatment of nuclear fragmentation. This early, transient expansion of the nuclear perimeter provides a clear morphological observation compatible with, but not direct evidence of, chromatin remodeling. Such macromorphological variations have been widely documented as phenotypic features following exposure to structural HDAC references, where histone hyperacetylation typically causes a relaxation of the tightly packaged nuclear matrix [48,49]. Prolonged exposure (24 h) led to classical apoptotic nuclear morphology, characterized by condensed, fragmented nuclei. Finally, compound **2** induced a significant accumulation of reactive oxygen species (ROS) in treated cells. Elevated ROS levels are widely recognized as a critical downstream mediator of HDACi-induced apoptosis, driving mitochondrial membrane depolarization and apoptotic signaling cascades [53,54].

While the present findings establish the potent enzymatic inhibitory activity and phenotypic cellular effects of compound **2**, a formal delineation of its complete selectivity profile across the wider HDAC family and the quantification of its therapeutic index remain the focus of ongoing work. In this preliminary hit-identification study, our experimental design was intentionally tailored to prioritize the uncovering of a novel chemical scaffold, its unique binding mode, and the characterization of its precise downstream mechanistic consequences, such as target-driven growth inhibition, ROS accumulation, and mitotic spindle failure. It is well established in early-stage phenotypic drug discovery that elucidating a new candidate’s specific mode of action provides far greater initial value for chemotype classification than collecting preliminary toxicity profiles in standard two-dimensional (2D) monolayer cultures of healthy cells.

Furthermore, a minor discrepancy was observed between the biochemical activity of compound **2** against isolated HDAC8 (97.56% inhibition at 1 µM) and its micromolar growth-inhibitory activity (GI_50_ values of 8.8–16 µM) across solid tumor cell lines. While this variation is common in target-directed screenings and likely reflects typical intracellular exposure factors, such as relative membrane permeability, non-specific binding to serum proteins in the culture medium, and intracellular metabolic dynamics, it underscores that single-concentration screening assays serve as primary energetic benchmarks rather than direct quantitative predictors of cellular potency [55]. To fully advance this non-hydroxamate chemotype along the drug development pipeline, the determination of a full concentration-response curve to establish an experimental IC_50_, alongside the benchmark of an HDAC8 reference inhibitor, will be implemented in subsequent optimization loops. Crucially, rather than relying on poorly predictive 2D screening frameworks, we firmly believe that true therapeutic windows, pharmacokinetics, and systemic safety parameters are accurately and meaningfully defined through sophisticated three-dimensional models and in vivo preclinical toxicological evaluations. These extensive in vivo characterizations lie beyond the intended scope of this initial hit-validation report and are planned as part of the subsequent lead-optimization phases.

Taken together, these findings identify compound **2** as a promising non-hydroxamate scaffold with promising inhibitory activity against HDAC8 and significant antiproliferative effects across diverse solid tumor models. The observed phenotypic responses, including early nuclear expansion, ROS accumulation, mitotic arrest, and apoptosis, are characteristic cellular hallmarks of HDAC pathway modulation, although their direct dependence on intracellular HDAC8 inhibition remains to be fully demonstrated. Overall, this study highlights shape-based virtual screening as an effective strategy for identifying non-hydroxamate hit scaffolds, providing a valuable starting point for the design of novel epigenetic anticancer agents.

## 4. Materials and Methods

### 4.1. In Silico Studies

Since the reliability of virtual screening strongly depends on the quality of the structural templates, the careful selection of appropriate PDB models was a key step in our workflow. We initially considered 52 human protein structures available in the Protein Data Bank [56]. To ensure reliability and relevance, several exclusion criteria were applied: models containing mutations, those linked to articles still marked as ‘to be published,’ and structures co-crystallized with redundant ligands or complex peptides were discarded. Following this refinement, the dataset was narrowed down to nine high-quality models: 1T64 [57], 1T67 [57], 1T69 [57], 1VKG [57], 1W22 [58], 3F07 [59], 3SFF [60], 3SFH [60], and 5FCW [43] (Table S1).

Among these nine models, we retained 3SFH and 3SFF because they feature amino acid-derived ligands that do not include the hydroxamic acid moiety, a key structural determinant in our study. The ligands from the remaining seven models (1W22, 1T64, 5FCW, 1T67, 1T69, 1VKG, and 3F07) were then analyzed using chemical fingerprinting to assess structural diversity. Initially, the ligands were prepared using LigPrep *v4.1* [61] by assigning the most probable protonation states at pH 7.4, preserving defined chiralities, adding hydrogen atoms, and performing energy minimization using the OPLS_2005 force field [62]. MACCS structural keys were generated for the compounds using Canvas *v3.5* [63], and the structural diversity of the dataset was evaluated based on the average Tanimoto similarity. Thus, we selected three ligands—1W22, 1T64, and 5FCW—as the most structurally diverse representatives for the subsequent virtual screening experiments. A detailed similarity matrix supporting this final selection is reported in Supplementary Table S2. Finally, we selected the following set of known HDAC8 inhibitors co-crystallized in five distinct PDB structures (PDB IDs: 1T64, 1W22, 5FCW, 3SFF, and 3SFH) (Figure 1). The receptor structures were energy-optimized by means of the Protein Preparation Wizard [64] tool implemented in Maestro *2018-1* (Schrödinger, LLC, New York, NY, USA) using OLPS_2005 as force field. Residual crystallographic buffer components and water molecules were removed, except for the one coordinated to the scissile carbonyl of acetyllysine, which plays a critical role in the first step of catalysis [59]. Hydrogen atoms were added, missing side chains and loops were modelled using the Prime module [65], and side chain protonation states were assigned based on physiological pH (7.4).

A library of 307,814 compounds was retrieved from the natural product-oriented subset of ZINC15 [41,42], which includes natural metabolites, natural product derivatives, and biogenic scaffolds, and was prepared using the LigPrep tool *v4.1*. Hydrogen atoms were added, salts were removed, and ionization states were assigned using Epik *v4.3* at pH 7.4. Subsequently, all compounds underwent energy minimization using the OPLS_2005 force field.

We implemented a shape-based virtual screening (SBVS) approach using the Phase [66] method to identify potential inhibitors from a natural compound database. This methodology relies on an initial rapid alignment of molecular shapes based on atom triplets, followed by a more detailed refinement and volume overlap scoring. The screening was performed by using the bioactive conformations of five co-crystallized ligands as reference structures.

To evaluate the shape similarity of the candidate molecules, we utilized a scoring function that combines both shape and atomic property features. For the shape comparison, we employed MacroModel [67] atom types. Prior to the screening, the ConfGen tool *v3.2*, integrated within the MacroModel suite, was used to generate a maximum of 100 conformers for each molecule in the database. The shape of each generated conformer was then aligned and compared to that of the query structures. A normalized shape similarity score, ranging from 0 (indicating complete dissimilarity) to 1 (representing identical shapes), was calculated for each conformer. Finally, we filtered the results to retain only those conformers with a shape similarity score of 0.7 or higher to at least one of the considered ligands.

We performed docking simulations using the HDAC8 crystal structure with the PDB code 5FCW. This model was selected after evaluating five potential candidates with a scoring algorithm designed to identify the most suitable structure for our study. The selection criteria prioritized recently deposited models with a crystallographic resolution better than 2 Å.

To identify the optimal structure, we applied a scoring algorithm based on the Cruickshank diffraction-component precision index (DPI) [68]. This index was calculated using the Blow [69] rearranged equation (Equation (1)), which incorporates several key crystallographic parameters to estimate coordinate precision and overall model quality.

(1) $$\mathrm{DPI}=\frac{1.28\;R_{\mathrm{free}}\sqrt{N_{i}^{3}}\sqrt{V_{a}}}{n_{\mathrm{obs}}^{5/6}}$$

Here, *R_free_* represents the cross-validation *R_factor_* computed from data not used for building or refining the X-ray model, *n_obs_* is the number of non-*R_free_* reflections, *N_i_* is the number of heavy atoms with occupancy equal to 1, and *V_a_* is the volume of the asymmetric unit cell.

The algorithm assigned a DPI score to each candidate model, with lower values indicating higher precision and better overall model quality. Among the five evaluated models, 5FCW exhibited the lowest DPI score, confirming it as the most suitable template for our docking simulations (Table S3).

Docking simulations were initiated using the crystal structure of HDAC8 complexed with a hydroxamic acid derivative (PDB ID: 5FCW). The docking grid was centered on the co-crystallized ligand within the refined binding site. To validate the reliability of the docking protocol, a redocking procedure was carried out using the Glide Standard Precision (SP) algorithm [70]. This method successfully reproduced the experimentally observed binding mode, yielding a root-mean-square deviation (RMSD) of 0.16 Å between the best-predicted pose and the co-crystallized ligand (Figure S7). Docking of the 9737 screened compounds was then conducted using Glide v.6.7 (SP mode), generating up to 10 poses per ligand. Compounds were ranked based on docking score and filtered using an energy cut-off established from the redocking benchmark. Subsequently, MM-GBSA calculations [65] were employed to estimate the thermodynamic stability of the ligand–receptor complexes. Both docking and MM-GBSA calculations were performed using the default settings implemented in the respective Schrödinger tools, without additional manual zinc-specific parameterization or explicitly imposed metal-coordination constraints; no additional manual adjustments were made to the protonation or tautomeric state of the benzamide zinc-binding group beyond those applied during the standard ligand preparation workflow. These candidates were clustered using Canvas *v3.5*, based on MACCS fingerprints and Tanimoto similarity. Final compound selection was based on visual inspection of the predicted binding modes within the HDAC8 active site, as well as the commercial availability of the candidates.

To evaluate the behavior of the most active inhibitor identified in this study, compound **2**, its complex with the HDAC8 enzyme was subjected to a 1000 ns (1 μs) molecular dynamics simulation, in parallel with a simulation of the HDAC8–co-crystallized ligand complex (PDB: 5FCW). The simulations were conducted using the Desmond *v5.3* [71] software package. The protein was modeled using the OPLS_2005 force field [62]. Both systems were prepared by solvating the complexes in an orthorhombic box filled with a 10 Å buffer of TIP4P water molecules, and Na^+^ ions were added to neutralize the overall system charge. Long-range electrostatic interactions were treated using the Particle Mesh Ewald (PME) method with a real-space cutoff of 10 Å.

The simulation started with an initial equilibration in the NVT ensemble at 10 K to relax the system. This was followed by a production phase consisting of three consecutive simulations under the NPT ensemble at 300 K and 1 bar, maintained using a Berendsen thermostat and barostat. The final production run was carried out for 1000 ns, with energy and trajectory data recorded every 1000 ps and a total of 1000 snapshots saved for subsequent analysis. A 2 fs integration time step was employed, with hydrogen bonds constrained using the SHAKE algorithm. The catalytic Zn^2+^ ion was parameterized using the standard non-bonded point-charge model, depicted as a spherical monoatomic centre with a formal charge of +2.0 *e*. Non-bonded interactions were calculated via standard Lennard-Jones 12-6 potentials coupled with long-range Coulombic electrostatics. No explicit harmonic bond or distance constraints were applied to the metal coordination sphere. Long-range electrostatics were calculated using the Particle Mesh Ewald (PME) method with a 10 Å cutoff. The ligand force field parameters were assigned using OPLS_2005. Partial atomic charges were evaluated via the CM1A-BCC charge model. All bond stretching, angle bending, and torsional parameters were retrieved from the OPLS_2005 parameter library based on assigned atom types. The 1 μs production run was conducted to evaluate the conformational persistence of the predicted docking pose, serving as qualitative structural support for the binding mode rather than an absolute measurement of binding thermodynamics.

### 4.2. Experimental Section

#### 4.2.1. Compounds Sourcing, Specifications, and Handling

All candidate molecules were purchased through Mcule (MCULE-5641977795, MCULE-2140072548, MCULE-6837965061, MCULE-6789463495, MCULE-7549319084, MCULE-3417253128, MCULE-2361206086).

According to the analytical documentation provided by the supplier (Mcule), compound **2** (MCULE-2140072548, internal batch ID: G16147) was supplied with a certified purity of 90%, as determined by ^1^H-NMR spectroscopy (300 MHz). The analytical ^1^H-NMR spectrum confirming the chemical identity and structure of compound **2** is provided in the Supplementary Materials (Figure S8).

Upon receipt, all tested compounds were stored as dry powders at −20 °C protected from light. Primary stock solutions were prepared in 100% high-purity dimethyl sulfoxide (DMSO) at a concentration of 10 mM, thoroughly vortexed to ensure complete dissolution, aliquoted, and maintained at −20 °C until use. Immediately prior to performing the enzymatic inhibition assays, working solutions were freshly prepared by serial dilution of the stock solutions into the assay buffer. The final DMSO concentration in all experimental reaction mixtures was strictly maintained at 1% (*v*/*v*) to avoid solvent interference with enzymatic activity.

#### 4.2.2. Enzymatic Validation of HDAC8 Inhibitory Activity

To validate the computational results, we determined the activity of HDAC8 using an enzyme assay kit (*Cayman Chemicals, 1180 East Ellsworth Road, Ann Arbor, MI 48108, USA*) according to the manufacturer’s protocols. HDAC8 activity was measured using a fluorometer (*Varioskan Lux, Thermo Fisher Scientific, Rodano, MI, Italy*) at excitation and emission wavelengths of 350 nm and 450 nm, respectively. To test the inhibitory activity of selected compounds, a concentration of 1 μM was used. The assay was conducted as a single-dose primary screening evaluation at a concentration of 1 μM to identify active hit scaffolds. Experimental IC_50_ determinations were not performed in this preliminary screening stage.

#### 4.2.3. Cell Culture

In this study we used different human carcinoma cell lines: K1 (papillary thyroid carcinoma cells), MDA-MB-157 and T-47D (breast cancer cells), Caco-2 and WiDr (colon cancer cells), A549 and SW1573 (Non-Small Cell Lung cancer), HeLa (cervical cancer cells) and MIA PaCa-2 (pancreatic ductal adenocarcinoma cells). Cells were cultured in DMEM or RPMI (*Thermo Fisher Scientific, Rodano, MI, Italy*) medium supplemented with 10% fetal bovine serum (FBS), 1% penicillin/streptomycin and 1% glutamine and maintained at 37 °C in a humidified atmosphere containing 5% CO_2_. The MDA-MB-157 (Cat. No. HTB-24), K1 (Cat. No. CRL-3604), and Caco-2 (Cat. No. HTB-37) cell lines were purchased directly from the American Type Culture Collection (*ATCC, Manassas, VA, USA*). Additionally, the human cancer cell lines A549 (Human lung adenocarcinoma; ATCC Cat. No. CCL-185, RRID:CVCL_0023); HeLa (Human cervical adenocarcinoma; ATCC Cat. No CCL-2, RRID:CVCL_0030); MIA PaCa-2 (Human pancreatic undifferentiated carcinoma; ATCC Cat. No. CRL-1420, RRID:CVCL_0428); SW1573 (also known as SW-1573) (Human alveolar exocrine lung carcinoma; ATCC Cat. No. CRL-2170, RRID:CVCL_1720); T-47D (Human mammary ductal carcinoma; ATCC Cat. No. HTB-133, RRID:CVCL_0553); and WiDr (Human colon adenocarcinoma; ATCC Cat. No CCL-218 or ECACC Cat. No. 85111501, RRID:CVCL_2760) were kindly provided by Prof. Godefridus J. Peters (*VUmc, Amsterdam, The Netherlands*). The cell lines were tested for their authentication by PCR profiling using short tandem repeats, which was performed by BaseClear (*Leiden, The Netherlands*). All experiments were performed using low-passage cells.

#### 4.2.4. Antiproliferative Assays

The anti-proliferative activity of the selected compound was tested by using 3-(4,5-dimethylthiazol-2-yl)-2,5-diphenyltetrazolium bromide (MTT) or Sulforhodamine B (SRB) assays.

For the MTT assay, MDA-MB-157, K1, Caco-2 and HeLa were seeded in 96-well plates at a density of 3.5 × 10^3^, 3 × 10^3^, 5 × 10^3^ or 2.5 × 10^3^, respectively. After 24 and 48 h of treatment with Compound **2**, 10 μL of MTT (0.5 mg/mL) was added to the cells and the solubilized product was quantified with a microplate spectrophotometer (*Varioskan Lux, Thermo Fisher Scientific Inc.*) at a wavelength of 540 nm and a reference wavelength of 690 nm. Results are expressed as percentages over untreated cells (vehicle), indicated as control.

For the SRB assay, we used our implementation of the protocol of the National Cancer Institute (NCI) of the USA. The following seeding densities (cells per well) were used: 2500 (A549, MIA PaCa-2, HeLa and SW1573) and 5000 (T-47D, WiDr). Samples for testing were dissolved initially in DMSO at 10 mM. For each test compound, the cells were exposed for a period of 48 h to serial decimal dilutions of the test compounds (0.001–100 μM) Afterwards SRB staining was performed. Briefly, cells were fixed with Trichloroacetic acid (TCA) and stained with SRB (0.4% *w*/*t*). Excess was washed with Acetic acid (8% *v*/*v*). Then SRB was dissolved using 10 mM Tris-base. Optical density of each well was determined at 530 and 620 nm using a PowerWave XS microplate reader. Dual wavelength was used to reduce optical interference caused by scratches, fingerprints, or other matter that absorbs light equally at both wavelengths. Values were corrected for background OD from wells containing only media. The antiproliferative activity, expressed as GI_50_ (50% growth inhibition), was calculated according to NCI formulas.

#### 4.2.5. Colony Formation Assay

HeLa cells were seeded (500 cells/well) onto 6-well plates. On the next day, compound **2** was added at a low (GI_50_/3) and a high (GI_50_) dose. Cells were exposed for seven days, then fixed with methanol and stained using crystal violet (0.5% *w*/*v* in deionized water). Colonies were counted using our in-house developed colony segmentation algorithm.

#### 4.2.6. Live Cell Imaging and Annexin Staining

HeLa cells were seeded (80,000 cells/dish) onto a 35 mm high glass-bottom μ-dish (*IBIDI, Munich, Germany*) and grown overnight. Then medium was changed to RPMI 1640 without phenol red, and compound **2** was added at 40 µM (3 × GI_50_) concentration. Then, the plates were placed in the CX-A label-free cell imaging system (*Nanolive S.A., Tolochenaz, Switzerland*). Images from a 236 μm × 236 μm area were taken every 5 min for 20 h. After image acquisition, cells were stained with Cy5 conjugated annexin (ab14150 from abcam^®^) as specified by the manufacturer. Fluorescence was imaged with the CX-A fluorescence module (*Gain 30, Intensity 10, Exposure 300 ms*). A surface of 400 μm × 400 μm was imaged. For quantitative studies, Eve segmentation and analysis software (*Nanolive S.A., Switzerland*) were used. The measurements were obtained for each cell at all imaging cycles for each treatment.

#### 4.2.7. Cell Migration Assay

A549 cells were seeded into a 24-well plate at a density of 30,000 cells/well. Cells were grown until 80–90% confluence was reached. Then a scratch was made in the culture using a 200 µL micropipette tip. Then culture medium was replaced with fresh medium (2.5% FCS) with or without compound **2**. Two doses of the candidate were used, 13 µM (high dose) and 4 µM (low dose). Afterwards pictures were taken with a brightfield microscope (*Axiovert 40 CFL, Zeiss, Germany*) at 5× magnification using the software ZEN 2012 (*blue edition v1.1.0.0*) at different time intervals (0, 6 and 24 h since the wound was made). TScratch [72] was used for the quantification of the wounded area, establishing manually the desired threshold.

#### 4.2.8. DAPI Staining

HeLa cells were seeded onto 6-well plates containing sterile coverslips at the bottom in a density of 100,000 cells/well. Cells were grown for 24 h, then compound was added at 20 µM (low dose) and 40 µM (high dose), and incubated for 24 h. Cells were fixed with 4% PFA (PBS) for 10 min at RT and washed in 50 mM NH4Cl for 10 min to reduce PFA background. Then cells were permeabilized with 0.1% Triton PBS solution for 10 min. Staining was performed using DAPI solution (1 µg/mL in PBS) for 10 min in darkness. Afterwards cells were rinsed 3 times with PBS and coverslips were mounted in a slide using Mowiol mounting medium and sealed using nail polish. Images of random image fields were taken using 40× magnifiers in a ZEISS Axiolab 5 epi-fluorescence microscope with excitation filter BP385/30. DAPI RFU was obtained by calculating the Integrated Density of the nuclei observed in at least 3 images per condition. Quantification of the mean fluorescence of DAPI-stained cells was performed using FIJI/ImageJ software *v1.54f* (*NIH, USA*).

#### 4.2.9. ROS Detection

Cells were seeded in black-walled, clear-bottom 96-well plates from Corning^®^. After 24 h, DCFDA was added at a final concentration of 40 μM, and cells were incubated for 30 min. Medium was then replaced with 100 μL of phenol red-free medium containing compound **2** at 40 µM or 10% H_2_O_2_. Fluorescence was measured at 492/520 nm using a Varioskan Lux multimode plate reader (Thermo Fisher Scientific). Wells were subsequently fixed with methanol and stained with 0.5% crystal violet, then solubilized in 1% SDS in PBS, and absorbance measured at 590 nm using a BioTek PowerWave XS plate reader for normalization.

#### 4.2.10. Statistical Analysis

Data from cell biological evaluations were obtained from a minimum of three independent biological experiments (*n* = 3), with each experiment performed using three technical replicates to ensure reproducibility. Quantitative results are expressed as mean ± standard deviation (SD). For assays involving the comparison of more than two groups—specifically the colony formation assay (Control, LD, HD), cell migration assay (Control, LD, HD), DAPI nuclear area kinetics (Control, 2 h, 4 h), and ROS detection/DCFDA fluorescence (Control, Compound **2**, H_2_O_2_)—statistical significance was determined using one-way analysis of variance (ANOVA) followed by Tukey’s multiple comparisons test. For the Annexin V staining assay, which evaluated two discrete conditions (Control vs. Compound **2**), a two-tailed Student’s *t*-test was applied. All statistical testing was performed using GraphPad Prism version 9.3.0 software, and differences were considered statistically significant at *p*-values < 0.05.

## Figures and Tables

**Table 1:** 2D structures, docking scores, and MM-GBSA binding free energy (ΔG_bind_) values of seven selected natural compounds identified through shape-based virtual screening and docking simulations. These compounds were chosen based on clustering results and commercial availability for experimental validation. All values are reported in kcal/mol.

Compound	2D Structure	D-Score *(kcal/mol)*	ΔG_bind_ *(kcal/mol)*
**1**	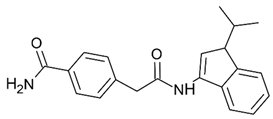	−8.30	−70.46
**2**	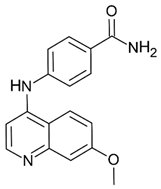	−8.94	−70.07
**3**	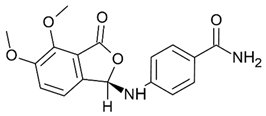	−8.70	−76.64
**4**	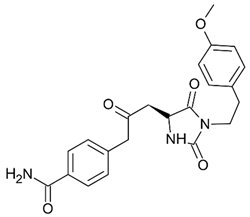	−8.23	−78.13
**5**	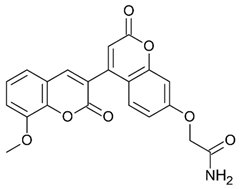	−8.29	−78.55
**6**	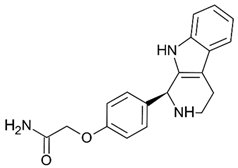	−8.21	−82.34
**7**	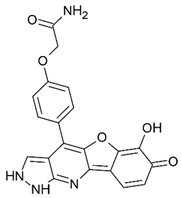	−9.37	−81.07

**Table 2:** GI_50_ (μM) values with high Standard Deviation for the in vitro screening against a panel of human solid tumor cell lines.

Tumor Cell Line						
	A549 (Lung)	MIA PaCa-2 (Pancreas)	HeLa (Cervix)	SW1573 (Lung)	T-47D (Breast)	WiDr (Colon)
**Compound 2**	13 ± 3.2	10 ± 2.5	8.8 ± 2.3	14 ± 0.83	16 ± 0.94	9.7 ± 1.6

## Data Availability

The original contributions presented in this study are included in the article and Supplementary Materials. Further inquiries can be directed to the corresponding author.

## Supplementary Materials

The following supporting information can be downloaded at https://www.mdpi.com/article/10.3390/ijms27219557/s1, References [73,74] are cited in Supplementary Material.

## Abbreviations

The following abbreviations are used in this manuscript:

HDAC	Histone Deacetylase
HAT	Histone Acetyltransferase
HDACis	Histone Deacetylase Inhibitors
ZBG	Zinc-Binding Group
CAP	Hydrophobic Cap Group
TSA	Trichostatin A
SAHA	Suberoylanilide Hydroxamic Acid
MM-GBSA	Molecular Mechanics-Generalized Born Surface Area
GI_50_	50% Growth Inhibition
LD	Low Dose
HD	High Dose
CTR	Control
MD	Molecular Dynamics
RMSD	Root Mean Square Deviation
RMSF	Root Mean Square Fluctuation
PDB	Protein Data Bank
SBVS	Shape-Based Virtual Screening
DPI	Diffraction-Component Precision Index
