# Supplementary material for: Discovery of Novel HDAC8 Inhibitors Through Shape-Based Virtual Screening: Experimental Validation and Characterization of a Promising Quinoline-Based Hit Scaffold
Source: Int J Mol Sci. 2026 Oct 26;27(21):9557. doi: 10.3390/ijms27219557 (PMC13763214; doi:10.3390/ijms27219557)
Supplement: Supplementary file 1 [file ijms-27-09557-s001.zip › ijms-4590488-supplementary.pdf]

Supplementary Information

Discovery of Novel HDAC8 Inhibitors through Shape-Based Virtual Screening: Experimental Validation and Characterization of a Promising Quinoline-Based Hit Scaffold

Adriana Gargano[1,2], Aday González-Bakker[3], Adrián Puerta[4], Valentina Maggisano[1], Stefania Bulotta[1], Roberta Rocca[1], José M. Padrón[3], Stefano Alcaro[1,2]

[1] Dipartimento di Scienze della Salute, Università "Magna Græcia" di Catanzaro, Campus Universitario "S. Venuta", Viale Europa, 88100 Catanzaro, Italy

[2] Associazione CRISEA – Centro di Ricerca e Servizi Avanzati per l'Innovazione Rurale, Località Condoleo, 88055 Belcastro (CZ), Italy

[3] BioLab, Instituto Universitario de Bio-Orgánica "Antonio González", Universidad de la Laguna, C/ Astrofísico Francisco Sánchez 2, 38206 La Laguna, Spain

[4] Institute of Biological and Chemical Systems - Functional Molecular Systems, Karlsruhe Institute of Technology (KIT), Eggenstein-Leopoldshafen 76344, Germany

TABLE OF CONTENTS

Figure S1. 2
Figure S2. 3
Figure S3. 3
Figure S4. 4
Figure S5. 4
Figure S6. .... **Error! Bookmark not defined.**
Figure S7. .... **Error! Bookmark not defined.**
Figure S8. .... **Error! Bookmark not defined.**
Table S1. 6
Table S2. 8
Table S3. 8
References .... **Error! Bookmark not defined.**

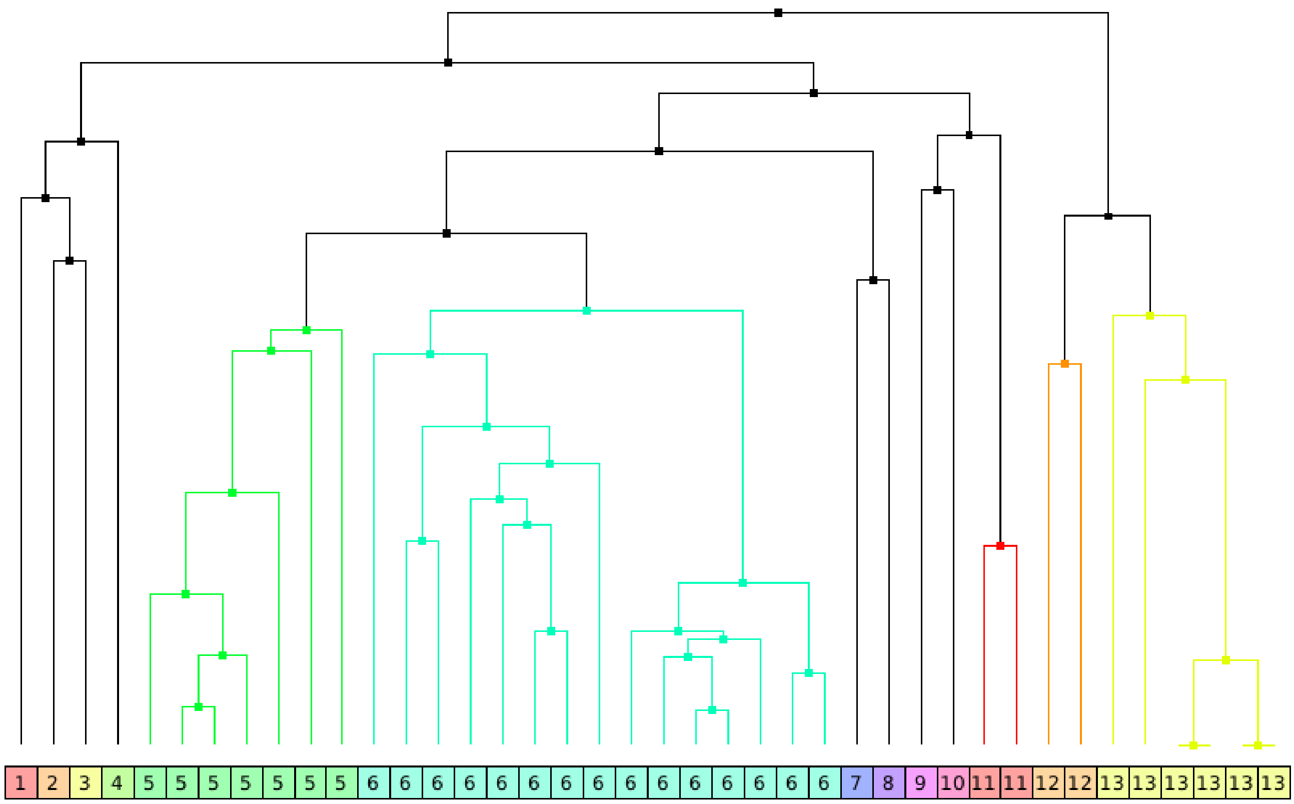

Figure S1. Hierarchical clustering dendrogram of the ligand dataset, grouped into 13 distinct clusters.

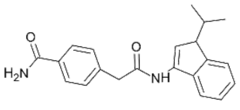

Cluster 1
ZINC000072325860
(Compound 1)

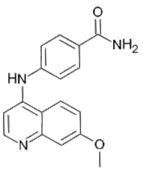

Cluster 2
ZINC000054202375
(Compound 2)

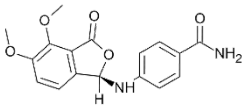

Cluster 3
ZINC000032124443
(Compound 3)

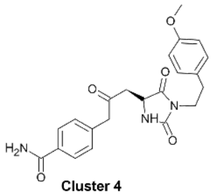

Cluster 4
ZINC000096114158
(Compound 4)

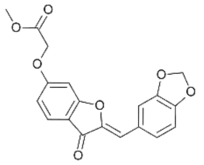

Cluster 5
ZINC000005956770
(Compound 5)

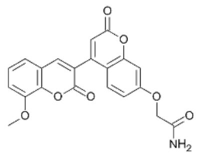

Cluster 6
ZINC000004340017
(Compound 6)

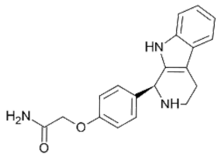

Cluster 7
ZINC000020112605
(Compound 7)

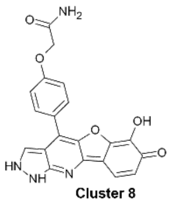

Cluster 8
ZINC000217671196
(Compound 8)

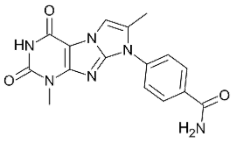

Cluster 9
ZINC000011693104
(Compound 9)

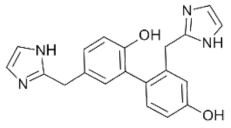

Cluster 10
ZINC000085996406
(Compound 10)

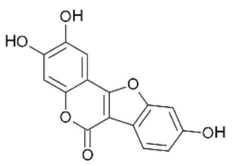

Cluster 11
ZINC000014778655
(Compound 11)

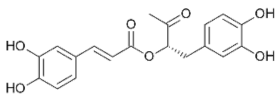

Cluster 12
ZINC000059587610
(Compound 12)

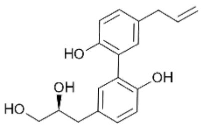

Cluster 13
ZINC000014585198
(Compound 13)

Figure S2. 2D chemical structures of the best compound representing each cluster. Each compound is labeled with its ZINC ID and the corresponding cluster to which it belongs.

Cell migration assay

Cell migration is a key process that leads to cancer cells invading the surrounding tissue and the local vasculature. Because of this, the discovery of small molecules affecting this process is desirable for cancer therapy as potential anti-metastatic compounds. In this study, the effects of **Compound 2** on cell migration capacity were evaluated through *In vitro* wound healing assay (Figure S3). A549 cancer cell line was used for this assay since it is a known model to study cell migration because of the motile and invasive properties of this cell line [73]. The results showed that the candidate does not affect the wound closure capacity of A549 cells. This is an unexpected result since it has been described that HDAC inhibitors affect cell migration_[74]. A plausible explanation is that under these conditions **Compound 2** did not affect the expression of migration-related genes.

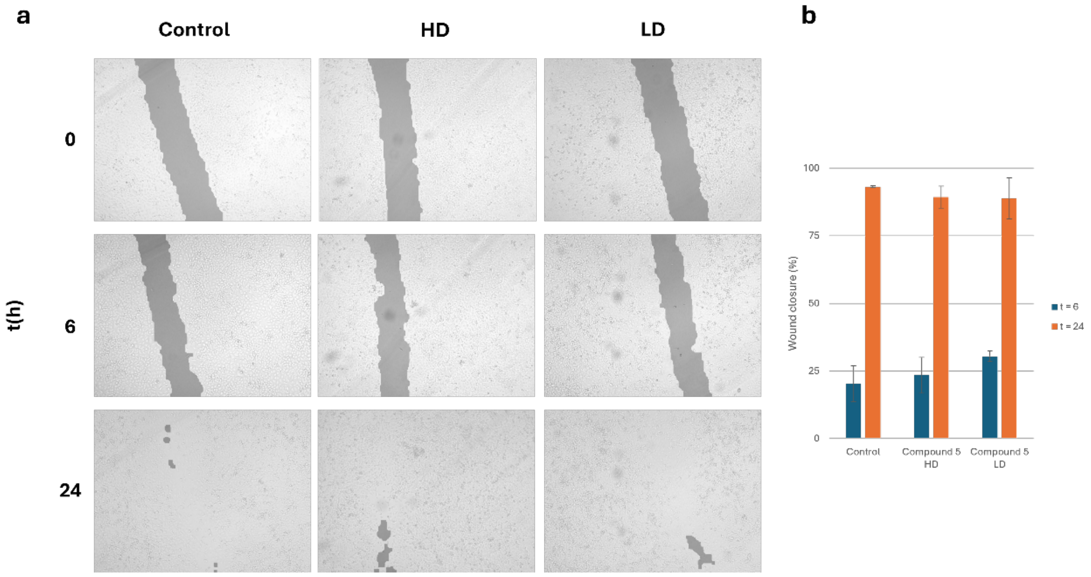

Figure S3. Effect of compound 2 on A549 cell migration in a wound-healing assay. (**a**) *In vitro* wound healing in A549 to compound **2** 13 µM (HD) or 4 µM (LD). (**b**) Graphical representation relative to wound closure. Data denote the mean ± SD of n = 3 independent biological experiments. Statistical profiling was performed utilizing one-way ANOVA followed by Tukey's multiple comparisons test to evaluate differences across all treatment groups at designated time points.

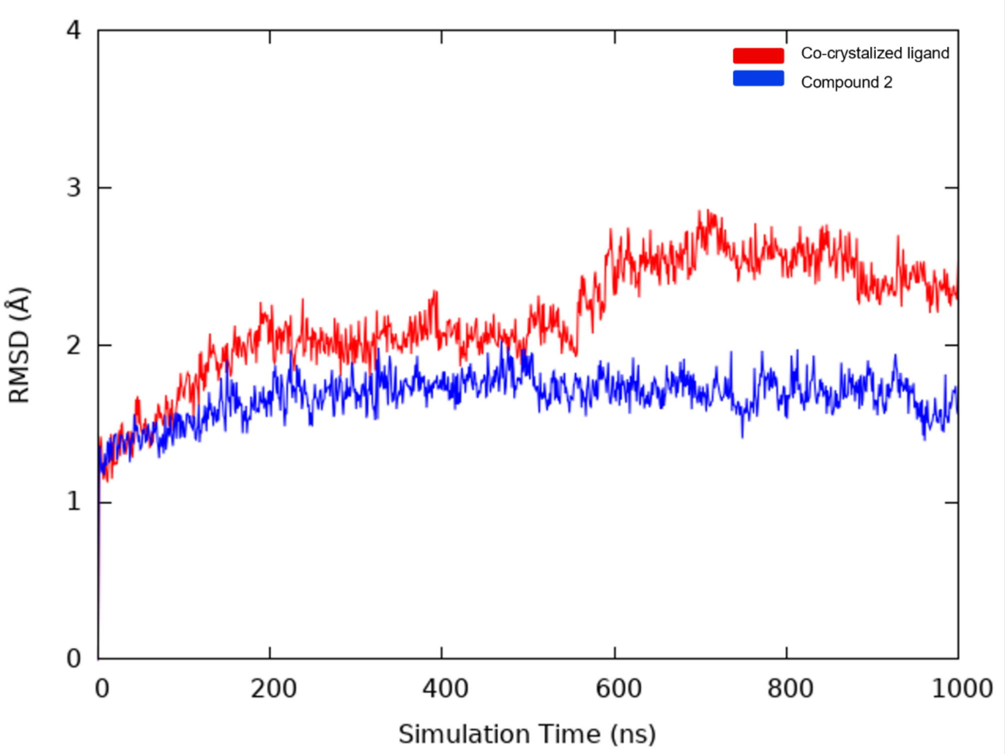

Figure S4. HDAC8 Backbone RMSD in Complex with the Co-crystallized Ligand and Compound 2. RMSD plot of the HDAC8 backbone in complex with the co-crystallized ligand (red line) and compound **2** (blue line) over 1 μs of molecular dynamics (MD) simulations. RMSD values were calculated by superimposing each trajectory frame onto the initial protein backbone structure, considering only the heavy atoms of the backbone.

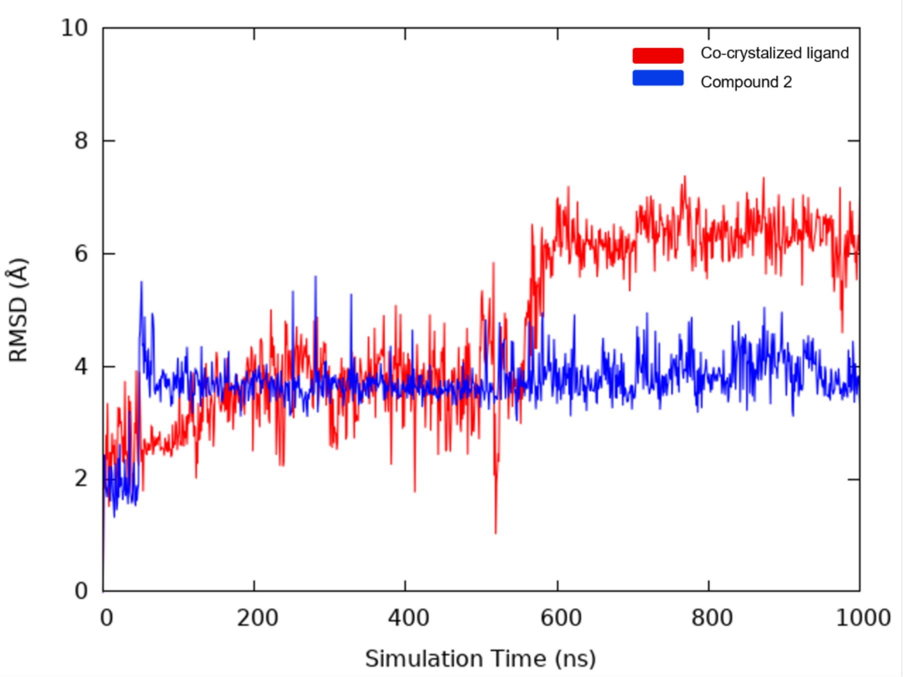

Figure S5. RMSD of the co-crystallized ligand and compound 2 within the HDAC8 binding site during 1000 ns molecular dynamics simulations. RMSD plot of the co-crystallized ligand (red line) and compound **2** (blue line) within the HDAC8 binding site over 1000 ns of molecular dynamics

(MD) simulations. RMSD values were calculated by superimposing each frame onto the protein backbone of the first frame, considering only the heavy atoms of the ligand.

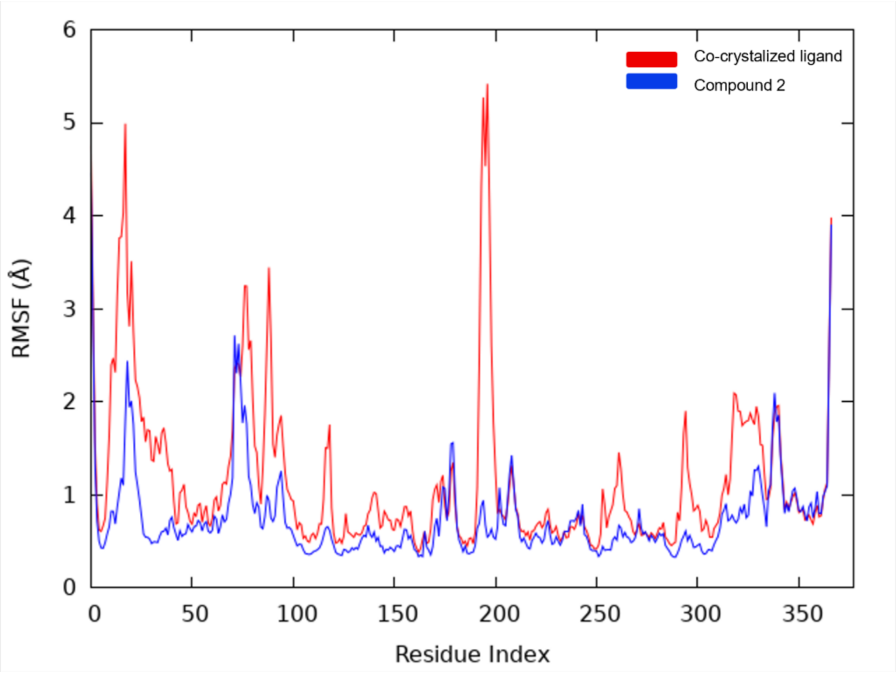

Figure S6. HDAC8 RMSF Profiles during 1 µs Molecular Dynamics Simulations. Plot of Root mean square fluctuations (RMSF) values over 1 µs of molecular dynamics (MD) simulations for the HDAC8–compound **2** complex (blue line) and the HDAC8–co-crystal ligand complex (red line).

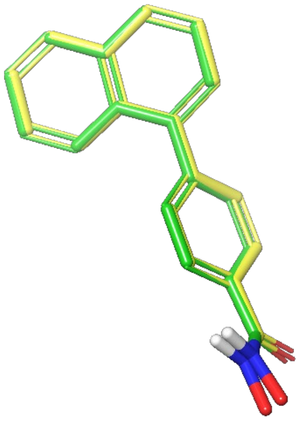

Figure S7. Redocking validation of the Glide SP docking protocol for HDAC8. Superposition of the experimentally determined binding pose of the co-crystallized ligand in the HDAC8 crystal structure (PDB ID: 5FCW) and the pose predicted by redocking. The redocking protocol reproduced the crystallographic binding mode with an RMSD of 0.16 Å. The crystallographic binding pose is represented in green sticks, while the reproduced docking pose is depicted in yellow sticks.

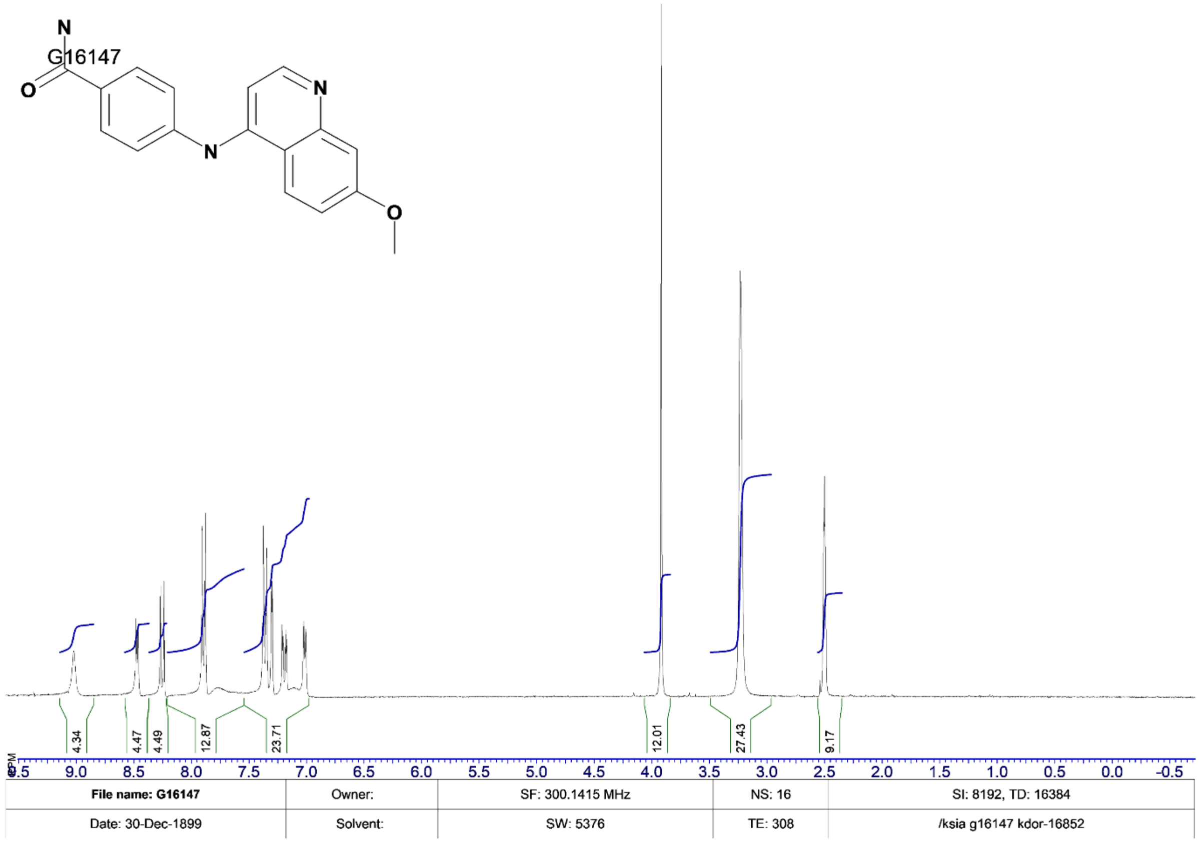

Figure S8. ^{1}H-NMR spectrum (300 MHz, DMSO-d_6) of compound 2. The spectrum confirms the molecular identity and structure of the compound (MCULE-2140072548, batch ID: G16147), in agreement with the batch purity specification (90%) provided by the commercial vendor (Mcule) and reported in Section 4.2 of the manuscript.

Table S1. Selected high-quality HDAC8 crystal structures and co-crystallized ligands. Selected high-quality HDAC8 crystal structures and their co-crystallized ligands. The table reports the 2D structures of the ligands, their IUPAC names, the corresponding PDB model, and the reference.

Chemical structure	IUPAC name	PDB model	Reference
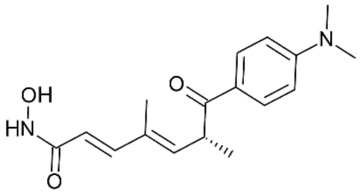	(2E,4E,6R)-7-[4-(dimethylamino)phenyl]-N-hydroxy-4,6-dimethyl-7-oxohepta-2,4-dienamide	1T64	[57]
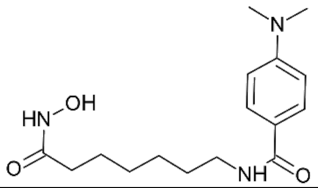	4-(dimethylamino)-N-[7-(hydroxyamino)-7-oxoheptyl]benzamide	1T67	[57]

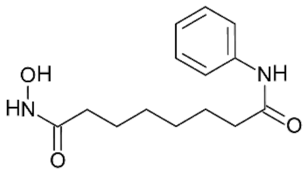	N'-hydroxy-N-phenyloctanediamide	1T69	[57]
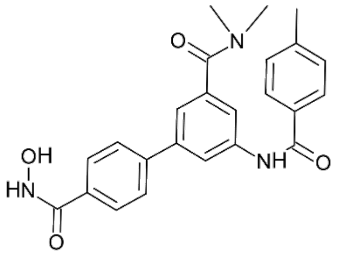	5-(4-Methyl-benzoylamino)-biphenyl-3,4'-dicarboxylic acid 3-dimethylamide-4'-hydroxyamide	1VKG	[57]
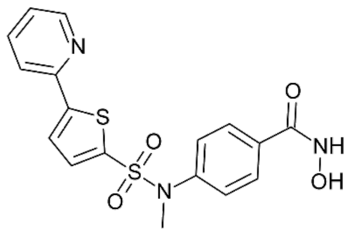	N-hydroxy-4-(methyl{[5-(2-pyridinyl)-2-thienyl]sulfonyl}amino)benzamide	1W22	[58]
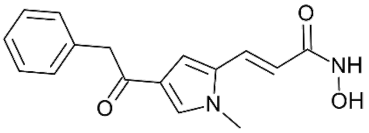	(2E)-N-hydroxy-3-[1-methyl-4-(phenylacetyl)-1H-pyrrol-2-yl]prop-2-enamide	3F07	[58]
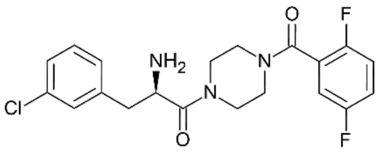	(2R)-2-amino-3-(3-chlorophenyl)-1-[4-(2,5-difluorobenzoyl)piperazin-1-yl]propan-1-one	3SFF	[60]
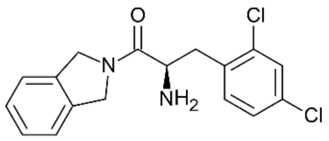	(2R)-2-amino-3-(2,4-dichlorophenyl)-1-(1,3-dihydro-2H-isoindol-2-yl)propan-1-one	3SFH	[60]
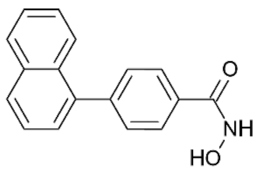	5-(4-Methyl-benzoylamino)-biphenyl-3,4'-dicarboxylic acid 3-dimethylamide-4'-hydroxyamide	5FCW	[43]

Table S2. Tanimoto similarity matrix of the co-crystallized ligands from the selected PDB models. Similarity matrix of the co-crystallized ligands from the selected PDB models. The values represent Tanimoto similarity coefficients, supporting the final selection of structurally different ligands for the virtual screening campaign.

	**1T64**	**1T67**	**1T69**	**1VKG**	**1W22**	**3F07**	**5FWC**
**1T64**	1	0,88	0,765957	0,895833	0,56338	0,77193	0,625
**1T67**	0,88	1	0,770833	0,86	0,637681	0,775862	0,6
**1T69**	0,765957	0,770833	1	0,744681	0,522388	0,614035	0,769231
**1VKG**	0,895833	0,86	0,744681	1	0,617647	0,694915	0,673913
**1W22**	0,56338	0,637681	0,522388	0,617647	1	0,621622	0,469697
**3F07**	0,77193	0,775862	0,614035	0,694915	0,621622	1	0,526316
**5FWC**	0,625	0,6	0,769231	0,673913	0,469697	0,526316	1

Table S3. DPI score of each PDB model.

PDB code	DPI
5FCW	0.105
3SFF	0.197
1T64	0.231
1W22	0.365
3SFH	0.504
